# Trehalose activates autophagy to alleviate cisplatin-induced chronic kidney injury by targeting the mTOR-dependent TFEB signaling pathway

**DOI:** 10.7150/thno.102559

**Published:** 2025-01-20

**Authors:** Jingchao Yang, Longhui Yuan, Lan Li, Fei Liu, Jingping Liu, Younan Chen, Ping Fu, Yanrong Lu, Yujia Yuan

**Affiliations:** 1 Department of Nephrology, National Health Commission (NHC) Key Laboratory of Transplant Engineering and Immunology, West China Hospital, Sichuan University, Chengdu 610041, China.; 2 Department of Nephrology, Institutes for Systems Genetics, Frontiers Science Center for Disease-Related Molecular Network, West China Hospital, Sichuan University, Chengdu 610041, China.; 3 Department of Nephrology, West China Hospital, Sichuan University, Chengdu 610041, China.

**Keywords:** autophagy, TFEB, mitochondrial dysfunction, chronic kidney disease, mTOR

## Abstract

**Rationale:** Cisplatin is a potent chemotherapeutic agent limited by significant nephrotoxicity. Multiple cycles of cisplatin administration are necessary to confer chronic disease. Autophagy is a lysosomal degradation pathway that enables the clearance and reuse of cytoplasmic components and is essential for maintaining the integrity and normal physiological function of tissues and organs. However, the precise role of autophagy in renal fibrosis has been controversial. Trehalose, a well-known autophagy inducer, plays a cytoprotective role under various stress conditions, such as oxidative damage, dehydration, and temperature changes. In this study, we established a model of cisplatin-induced chronic kidney disease (CKD) and human renal tubular epithelial cells (HK2) injury to investigate the nephroprotective effects of trehalose on cisplatin-induced CKD and the underlying mechanisms involved.

**Methods:** Firstly, we measured the role of autophagy in cisplatin-induced injury models both *in vivo* and *in vitro* by western blot and immunofluorescence staining, combined with transcriptomics. Then, biomedical, cellular, and molecular approaches were utilized to evaluate the potential protective effect of trehalose intervention in regulating autophagy. Mechanistically, we performed this study using proximal tubular epithelial cells-specific transcription factor EB (TFEB) knockout mice and TFEB small-interfering RNA technology to determine whether TFEB deficiency affects the pharmacological effected of trehalose in cisplatin-induced injury models.

**Results:** Due to the activation of autophagy, trehalose inhibited mitochondrial dysfunction (mitochondrial fragmentation, depolarization, reactive oxygen species) and cellular senescence induced by cisplatin both *in vitro* and *in vivo*. Moreover, renal dysfunction, pathological changes and fibrosis were alleviated in CKD mice after trehalose treatment. Mechanistic investigations revealed that trehalose accumulated in lysosomes and inhibited mTORC1 activity, which triggered TFEB and TFEB-mediated autophagy. In addition, siRNA-mediated knockdown of TFEB in HK2 cells or renal proximal tubular epithelial cells-specific (TECs-specific) TFEB deficiency in mice markedly abolished the beneficial effects of trehalose.

**Conclusion:** Our findings suggested that trehalose induced autophagy to alleviate cisplatin-induced chronic kidney injury by targeting the mTOR-dependent TFEB signaling pathway.

## Introduction

Acute kidney injury (AKI) is a syndrome characterized by rapid deterioration of renal function. The pathological process of AKI can be summarized into four grades: mild, self-limiting, severe and persistent 1. At present, increasing evidence has shown that AKI is one of the main factors involved in the development of CKD 2. The process from AKI to CKD is known as the "AKI-CKD transition", which is also associated with high cardiovascular risk and the development of end-stage renal disease 3.

Renal tubular epithelial cell injury is one of the most important events in the process of AKI and CKD 4, 5. Renal tubular epithelial cells undergo repair and regeneration after AKI. Mild renal injury induces complete repair of renal tubular epithelial cells, but severe renal injury leads to maladaptive repair. During this process, renal tubular epithelial cells produce profibrotic factors, leading to persistent tubular interstitial inflammation, fibroblast proliferation and excessive deposition of the extracellular matrix. Therefore, maladaptive repair is a link between AKI and CKD 6.

Autophagy is a lysosomal degradation pathway in which cytoplasmic components are cleared and reused. The autophagic process consists of several steps, namely initiation, nucleation, expansion, fusion and degradation. The cytosolic LC3-I convers to membrane-bound LC3-II is indicative of autophagy induction and autophagosome formation 7. The sequestosome 1/p62-associated ubiquitin system, the major factor of final degradation, which functions as a selective autophagy adaptor by simultaneously capturing ubiquitinated cargo and interacting with LC3-II 8. Autophagy is a dynamic process and therefore the turnover of proteins (such as LC3 II, P62) has to be artificially blocked in order to adequately represent the activity of autophagy. Bafilomycin A1 (Baf) and hydroxychloroquine (HCQ) are commonly used autophagy inhibitors that primarily block lysosomal degradation. Their use leads to an accumulation of autophagy-related proteins, specifically LC3 II and p62 9.

The basic level of autophagy is essential for maintaining renal homeostasis, structure and function. Autophagy is activated in proximal tubules as a protective mechanism in AKI, but sustained activation of autophagy induces phenotypic changes in proximal tubules, which may lead to poor adaptive repair and interstitial fibrosis and promote the transition from AKI to CKD 10. In contrast to the findings of this study, renal fibrosis was reduced in mice in which autophagy-related 7 was knocked out in the proximal tubules after UUO 11. Thus, the precise role of autophagy in renal fibrosis has been controversial.

Trehalose is a nonreducing disaccharide that exists in bacteria, fungi, insects, plants and other organisms 12. In the past decade, the autophagy-inducing properties of trehalose, which can activate autophagy in different types of mammalian cells in culture, have been the subject of many studies. According to previous studies, trehalose can induce autophagy and slow disease progression in disease models such as those of Parkinson's disease and Huntington's disease 13.

Therefore, in this study, we used cisplatin-induced CKD mouse model and HK2 injury model to explore the role of autophagy and further studied the molecular mechanism of autophagy.

## Results

### Trehalose activates autophagy and alleviates CKD

**Figure 1:**
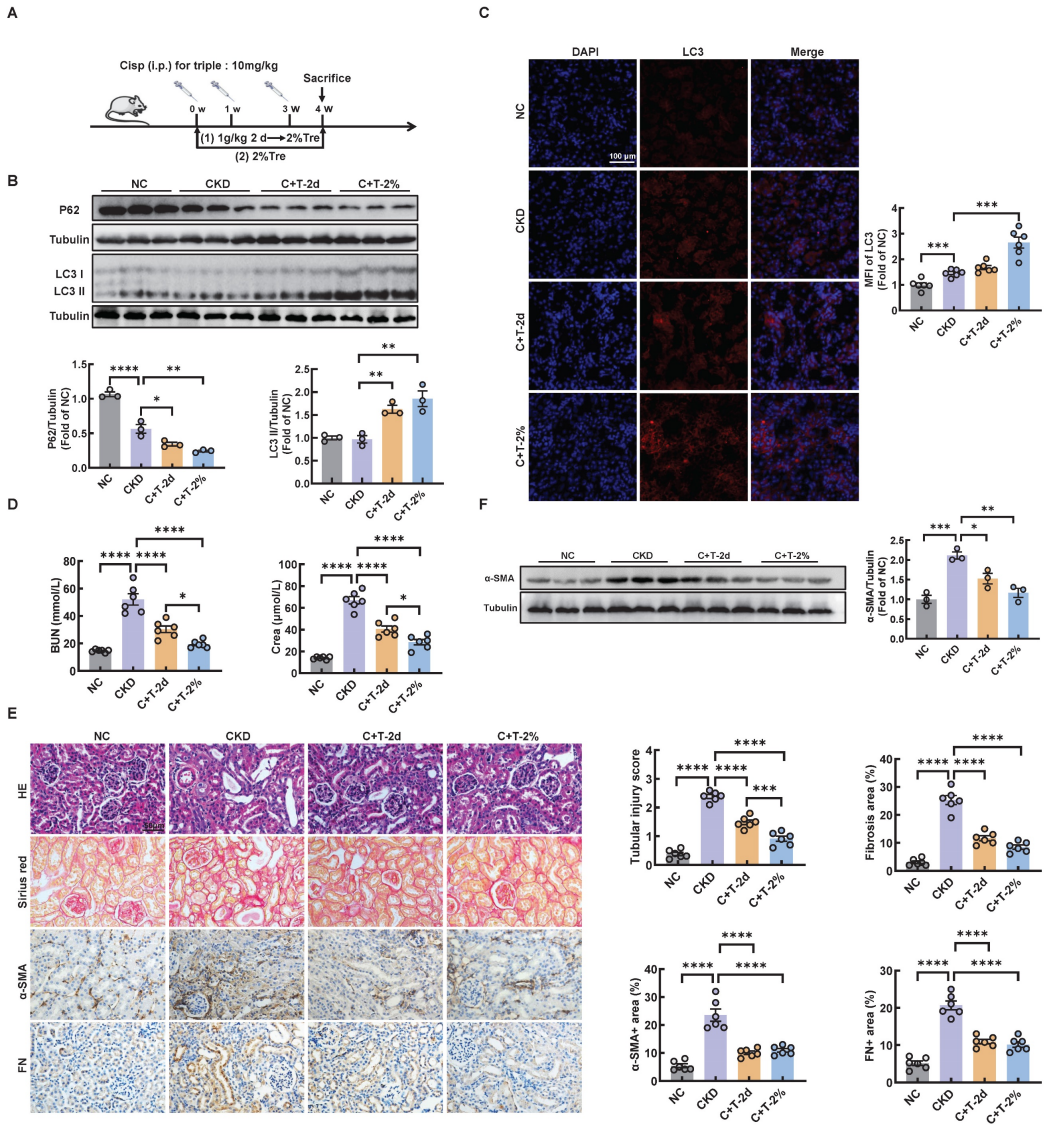
** Trehalose activates autophagy and alleviates CKD.** (A) Illustration of trehalose treatment in cisplatin-induced CKD mice. First, the mice were injected intraperitoneally at a dose of 1 g/kg body weight, and after 48 hours, the treatment group was orally administered 2% w/v solution for 4 weeks. Second, the mice were orally administered trehalose diluted in distilled water in the drinking water at a final concentration of 2% w/v for 4 weeks. (B) Western blot and quantitative analyses of P62 and LC3 II. Tubulin was used as the loading control. (C) Representative immunofluorescence images and quantification of LC3 (red) in kidney sections. Scale bar, 100 μm. (D) The serum levels of BUN and CREA in the mice. (E) Representative images of hematoxylin-eosin (HE) and sirius red-staining (Sirius red), immunohistochemical staining and quantification of α-SMA and fibronectin (FN) in paraffin-embedded kidney sections. Scale bars, 50 µm. (F) Western blot and quantitative analyses of α-SMA. Tubulin was used as the loading control. n = 6 mice per group were used to analyze the results. Data are shown as the means ± SD from at least three independent experiments and analyzed by one-way ANOVA with Tukey's test. *P < 0.05, **P < 0.01, ***P < 0.001, ****P < 0.0001. (NC, normal control; CKD, cisplatin-induced CKD mice; C+T-2d, trehalose at 1 g/kg of body weight was injected intraperitoneally, and 48 hours later, 2% w/v solution was added to the treatment group; C+T-2%, trehalose diluted in drinking distilled water at a final concentration of 2% w/v solution).

Trehalose is widely recognized as an activator of autophagy. To validate the effect of trehalose *in vitro*, we incubated cisplatin-treated HK2 cells with or without trehalose. Here, we found that autophagy was activated in cisplatin-treated HK2 cells (Figure S1A-C). Similar to our previous study in cisplatin-induced model of AKI 14, we demonstrated that autophagy was also activated in cisplatin-induced model of CKD (Figure S2A-C). We confirmed by western blotting that trehalose treatment further increased the expression of microtubule-associated protein 1 light chain 3-II (LC3 II), a biochemical hallmark of autophagy, compared with that in cisplatin-treated HK2 cells (Figure S3A). Similar to the findings in HK2 cells, we examined the effect of trehalose treatment on mice with cisplatin-induced CKD. We first evaluated the safety of trehalose in mice. As shown in Figure S4A, trehalose treatment for 4 weeks had no effect on blood urea nitrogen (BUN) and serum creatinine (Crea) levels (Figure S4A-B). Moreover, no pathological changes in the structure of the kidney and liver were observed (Figure S4C). These data demonstrated that trehalose did not cause nephrotoxicity and hepatotoxicity in mice. Then, the mice were intraperitoneally injected with cisplatin and administered trehalose at the same time. Trehalose was at 1 g/kg of body weight via intraperitoneal injection, and 48 hours later, 2% w/v solution was administered to the treatment group (C+T-2d) or orally administered diluted in drinking distilled water at a final concentration of 2% w/v solution (C+T-2%) (Figure 1A). We validated the increase in LC3 II and decrease in P62 after treatment with trehalose by western blotting (Figure 1B). Consistent with these findings, immunofluorescence revealed that compared with those in the cisplatin group, the expression of LC3 in the trehalose-treated group was greater (Figure 1C). Taken together, these results indicated that trehalose further activated autophagy.

To clarify the therapeutic role of trehalose, we further investigated whether trehalose could ameliorate kidney injury in CKD mice. Notably, cisplatin-induced kidney injury was alleviated after the administration of trehalose, as indicated by decreased levels of BUN and Crea, and reduced pathological damage (Figure 1D-E). In addition, interstitial fibrosis in CKD mice was also suppressed after trehalose treatment, further supporting the protective role of trehalose in CKD mice (Figure 1E-F). Collectively, these data suggest that trehalose activates autophagy and alleviates CKD.

### Trehalose attenuates mitochondrial dysfunction

**Figure 2:**
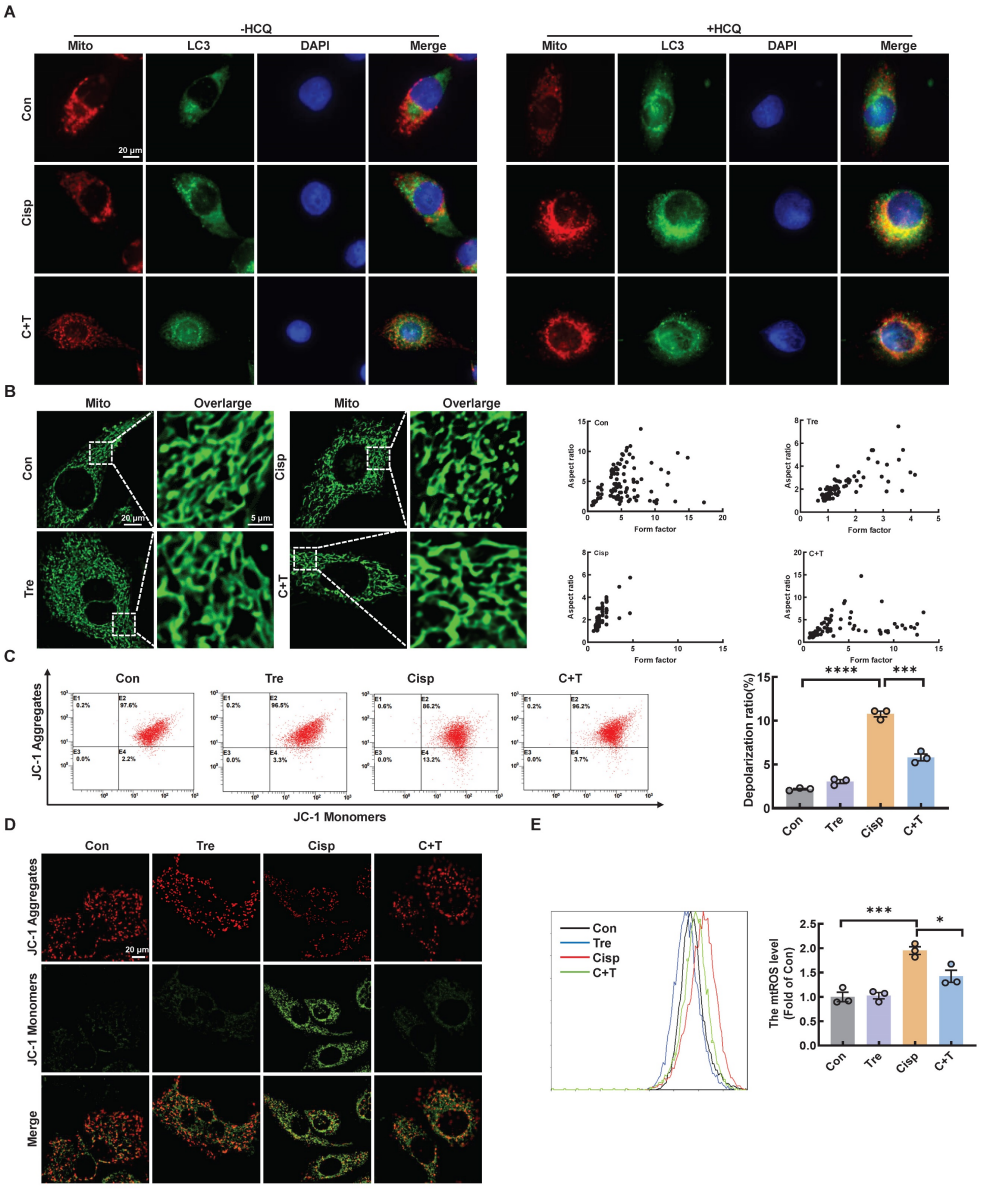
** Trehalose attenuates mitochondrial dysfunction *in vitro*.** (A) Representative images showing colocalization of LC3 (green) with mitochondria (red) treated with or without hydroxychloroquine (HCQ, 20 µM). Scale bar, 20 μm. (B) Representative immunofluorescence images of mitochondrial morphology (green) in HK2 cells loaded with MitoTracker Green. Scale bars, 20 µm and 5 µm (C) Mitochondrial membrane potential was measured with JC-1 and quantified by flow cytometry. (D) Detection of JC-1 aggregates (red) and monomers (green) in HK2 cells by confocal fluorescence microscopy. Scale bar, 20 μm. (E) Mitochondrial ROS (mtROS) was measured by flow cytometry following incubation with MitoSOX. Data are shown as the means ± SD from at least three independent experiments and analyzed by one-way ANOVA with Tukey's test. *P < 0.05, ***P < 0.001, ****P < 0.0001. (Con, control; Tre, trehalose; Cisp, cisplatin; C + T, cisplatin + trehalose).

**Figure 3:**
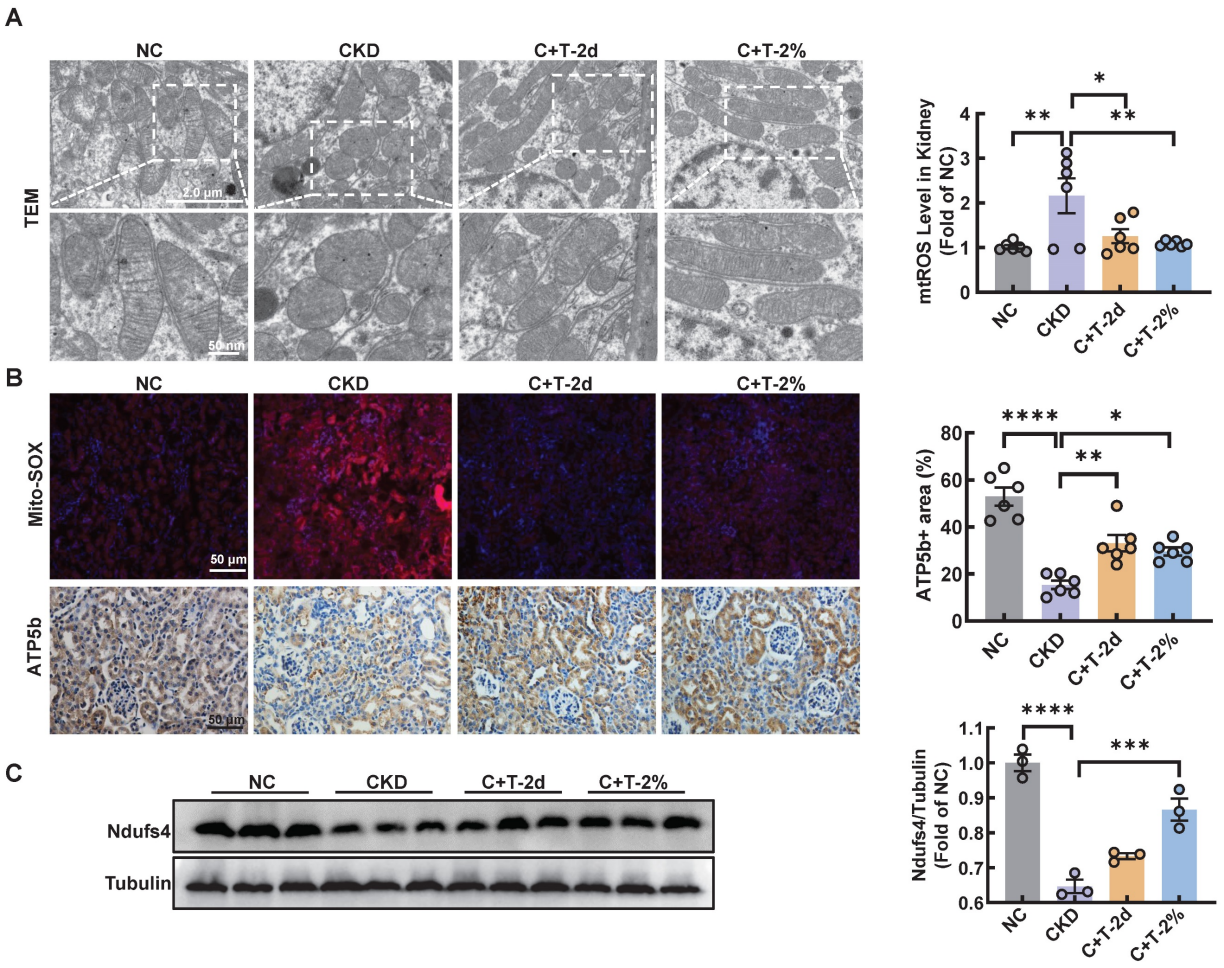
** Trehalose attenuates mitochondrial dysfunction *in vivo*.** (A) Representative TEM micrographs of mitochondria in kidney sections from each group. Scale bar, 2 μm and 50 nm. (B) Representative images and quantification of mitochondrial ROS (mtROS) (red) in kidney sections. Scale bars, 50 µm. Immunohistochemical staining and quantification of ATP5b expression in kidney sections. Scale bars, 50 µm. (C) Western blot and quantitative analyses of Ndufs4. Tubulin was used as the loading control. n = 6 mice per group were used to analyze the results. Data are shown as the means ± SD from at least three independent experiments and analyzed by one-way ANOVA with Tukey's test. *P < 0.05, **P < 0.01, ***P < 0.001, ****P < 0.0001. (NC, normal control; CKD, cisplatin-induced CKD mice; C+T-2d, 1 g/kg body weight trehalose was injected intraperitoneally, and 48 hours later, 2% w/v solution was added to the treatment group; C+T-2%, trehalose diluted in distilled water in the drinking water at a final concentration of 2% w/v solution).

Recent reports have shown that cisplatin accumulates in the mitochondrial matrix and causes mitochondrial dysfunction, ultimately resulting in proximal tubular cell death 15. Mitochondrial damage plays a major role in fibrosis, this damage can activate autophagy, and autophagy can also clear damaged mitochondria and maintain mitochondrial homeostasis. Mitophagy, a type of selective autophagy, is pivotal for mitochondrial maintenance. The above data demonstrated that the LC3 II level was increased in HK2 cells treated with cisplatin, while the P62 level was decreased (Figure S1A). According to the guidelines for the use and interpretation of assays for monitoring autophagy (4rd edition), the accumulation of LC3 Ⅱ as it may relate to either the induction of autophagy or the inhibition of autophagic degradation 16, 17. To distinguish the two possibilities, we treated HK2 cells with HCQ, a lysosomal acidification inhibitor, the colocalization of LC3 and mitochondria in cisplatin-treated HK2 cells increased greatly after trehalose, indicating that mitophagy was activated (Figure 2A). To assess the protective effect of mitophagy on mitochondrial dysfunction, we examined mitochondrial structure and function in a cisplatin-induced injury model after trehalose treatment. We first observed the mitochondrial structure in the cisplatin-induced group, which exhibited mitochondrial fragmentation, while mitochondrial impairment was significantly suppressed after trehalose treatment (Figure 2B). Moreover, compared with those in the cisplatin-induced group, the depolarization of the membrane potential and the overproduction of mtROS were reversed in the trehalose-treated group (Figure 2C-E). In line with the results in cell culture models, we also detected the effect of trehalose in mice with cisplatin-induced CKD. Notably, compared with those in CKD mice, we found that the number of swollen mitochondria, vacuolization, and ridge breakage were greatly inhibited by trehalose intervention (Figure 3A). Moreover, compared with those in cisplatin-induced CKD mice, mtROS overproduction in the kidney markedly decreased after trehalose administration, as assessed by mitoSOX fluorescence (Figure 3B). Similarly, we validated the increase in the expression of mitochondrial transport chain complex proteins (ATP5b and Ndufs4) by immunohistochemistry and western blotting (Figure 3B-C). Taken together, these results demonstrate that trehalose attenuates mitochondrial dysfunction.

### Trehalose ameliorates cisplatin-induced senescence

**Figure 4:**
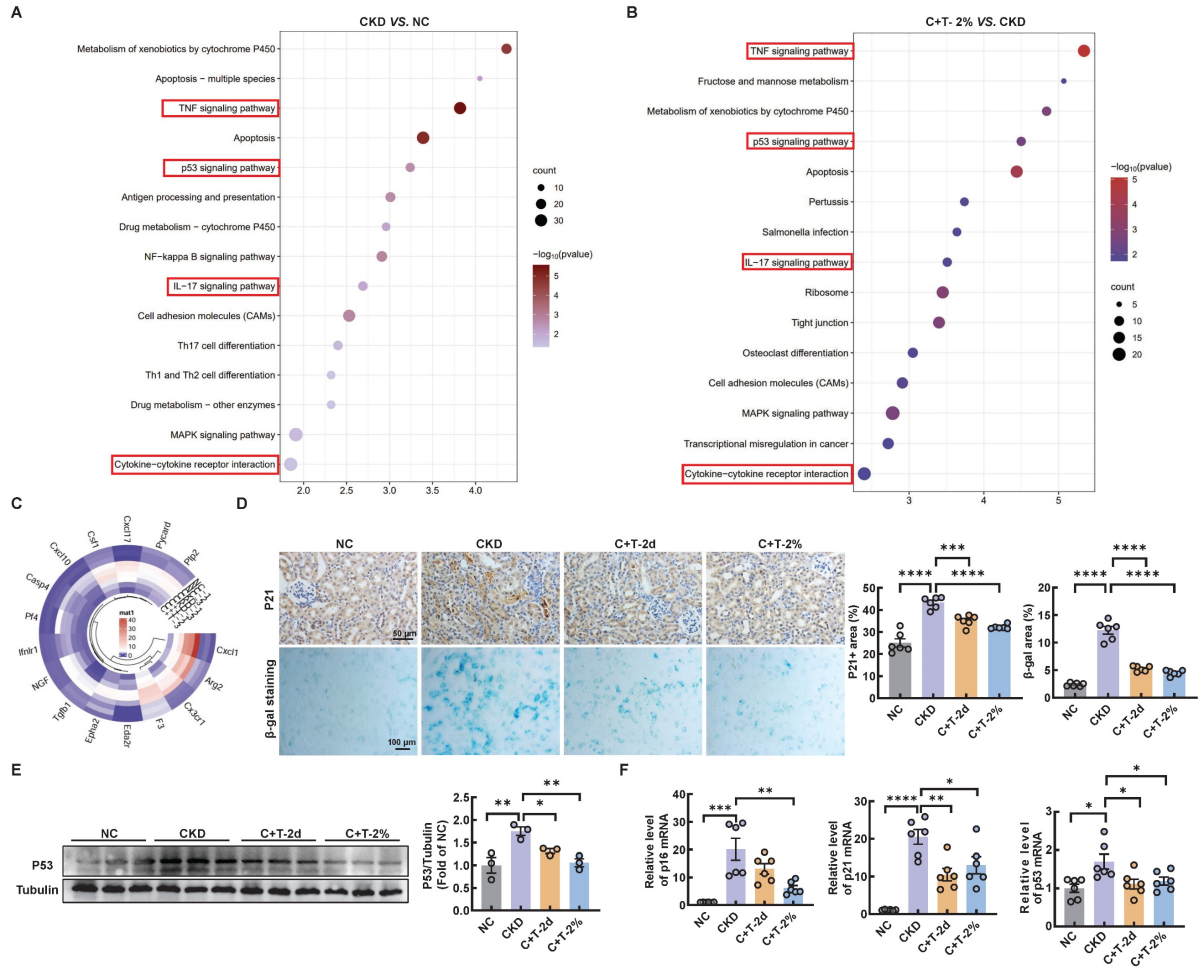
** Trehalose ameliorates cisplatin-induced senescence *in vivo*.** The kidneys of control and cisplatin-induced CKD mice were subjected to RNA-seq, and the differentially increased genes were enriched and subjected to (A) KEGG pathway enrichment analysis. Cisplatin-induced CKD mouse kidneys and trehalose-treated mouse kidneys were subjected to RNA-seq, and differentially downregulated genes were enriched and subjected to (B) KEGG pathway enrichment analysis. (C) Circular heatmap of SASP-related genes in kidney sections associated with the 3 different groups. (D) Representative images of immunohistochemical staining of P21. Scale bar, 50 μm. Renal tissue was stained for SA-β-Gal activity. Scale bar, 100 μm. (E) Western blot and quantitative analyses of P53. Tubulin was used as the loading control. (F) The mRNA levels of p16, p21 and p53 in the 4 different groups. n = 6 mice per group were used to analyze the results. Data are shown as the means ± SD from at least three independent experiments and analyzed by one-way ANOVA with Tukey's test. *P < 0.05, **P < 0.01, ***P < 0.001, ****P < 0.0001. (NC, normal control; CKD, cisplatin-induced CKD mice; C+T-2d, 1 g/kg body weight trehalose was injected intraperitoneally, and 48 hours later, 2% w/v solution was added to the treatment group; C+T-2%, trehalose diluted in drinking distilled water at a final concentration of 2% w/v solution).

Mitochondrial dysfunction is a contributing factor to cellular senescence, which in turn can further exacerbate mitochondrial impairment in a feedback loop way. Previous studies have shown that renal tubular cell senescence is an early stage in renal fibrosis 18, 19. Senescent cells can secrete multifarious factors, collectively known as the SASP, which activate neighboring cells to induce and accelerate organ fibrosis, especially renal fibrosis 20-22. To determine whether cisplatin induces senescence in renal tubular cells *in vitro*, senescence-associated β-galactosidase activity was increased compared with that in the control group, while a significant decrease was observed after trehalose treatment (Figure S5A). Similarly, the increased mRNA levels of senescence related genes (p21, and p53) and SASP-related genes (IL-1β, and TGF-β) induced by cisplatin were significantly reduced after trehalose treatment. With regard to the mRNA levels of p16 and IL-6, a similar downward trend was observed although there was no statistical difference (Figure S5B). We conducted further analysis using RNA sequencing *in vivo*. KEGG pathway analysis revealed that compared with control mice, CKD mice were enriched in aging and inflammatory signaling pathways, which were reversed after trehalose intervention (Figure 4A-B). In addition, the expression of SASP-related genes, such as CXCL1, Arg2, and CX3CR1, was increased in the CKD mice, while decreased with the treatment of trehalose (Figure 4C). Furthermore, in line with the findings* in vitro*, we consistently demonstrated that trehalose ameliorated cisplatin-induced senescence, as validated by immunohistochemistry, β-gal staining, western blotting and RT-PCR (Figure 4D-F). Taken together, our data confirm that trehalose attenuates senescence both* in vitro* and *in vivo*.

### D+Q treatment ameliorates renal injury in a murine model of multiple cisplatin treatments

**Figure 5:**
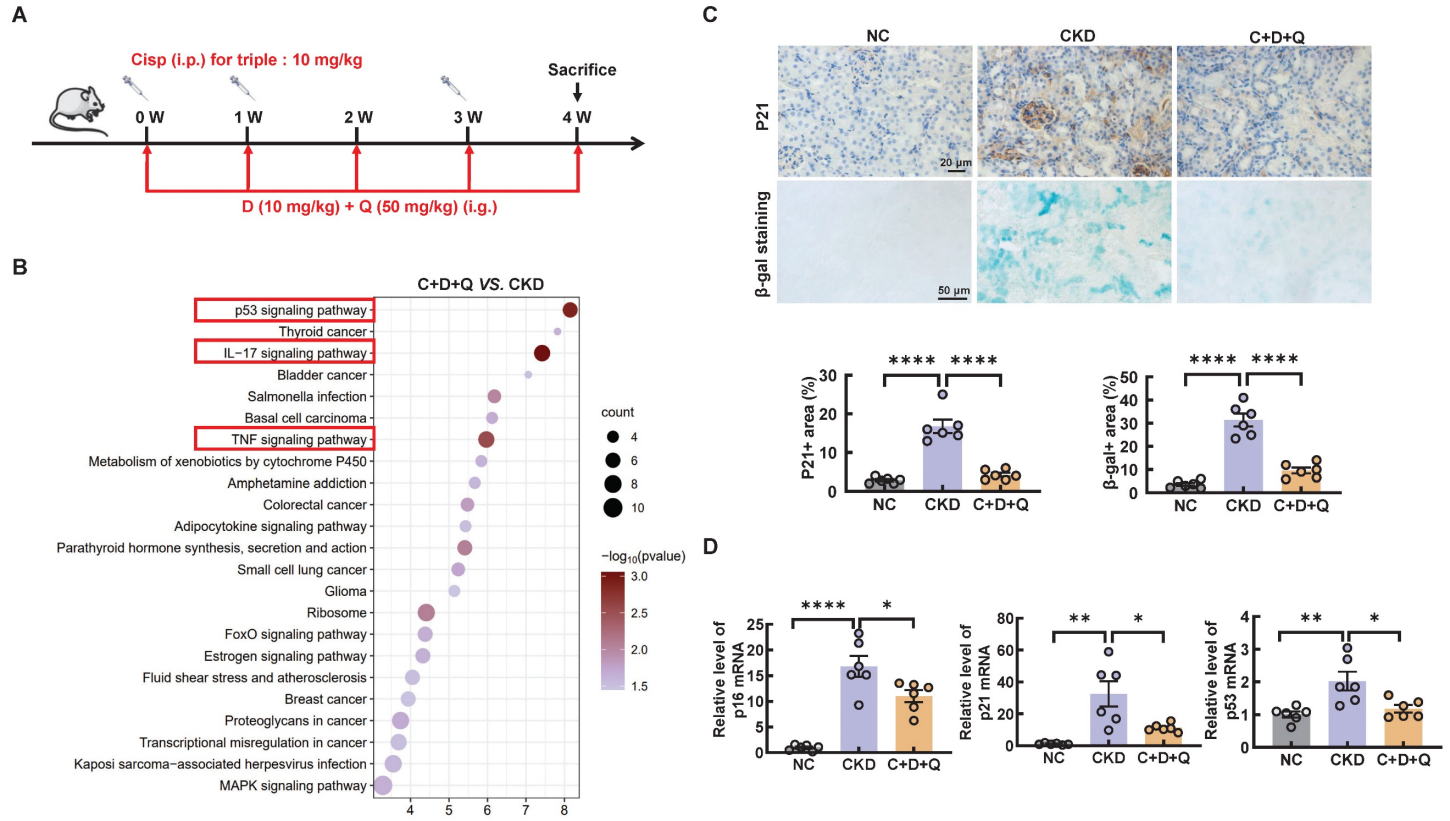
** D+Q treatment ameliorates renal senescence in multiple cisplatin-induced CKD mice.** (A) CKD mice were orally administered dasatinib (5 mg/kg) plus quercetin (50 mg/kg) (dasatinib + quercetin, D+Q) once a week. Mice were killed at 4 weeks after the first cisplatin injection. (B) KEGG pathway enrichment analysis of downregulated genes between the cisplatin-induced CKD group and the D+Q intervention group. (C) Representative images of immunohistochemical staining of P21. Scale bar, 20 μm. Renal tissue was stained for SA-β-Gal activity. Scale bar, 50 μm. (D) The mRNA levels of p16, p21 and p53 in the 3 different groups. n = 6 mice per group were used to analyze the results. Data are shown as the means ± SD from at least three independent experiments and analyzed by one-way ANOVA with Tukey's test. *P < 0.05, **P < 0.01, ****P < 0.0001. (NC, normal control; CKD, cisplatin-induced CKD mice; C+D+Q, cisplatin-induced CKD mice were orally administered D plus Q).

Senolytics refers to the selective elimination of senescent cells 23. Of note, dasatinib (D) and quercetin (Q) administered in combination are currently the most studied anti-aging drugs. To further investigate the role of cellular senescence in cisplatin-induced CKD, we subjected cisplatin-induced CKD mice to oral gavage once a week for up to 4 weeks (Figure 5A). To determine the underlying causes of these phenomena, we performed RNA-sequencing analysis and found decreased enrichment of senescence and inflammation signaling pathways (Figure 5B).

**Figure 6:**
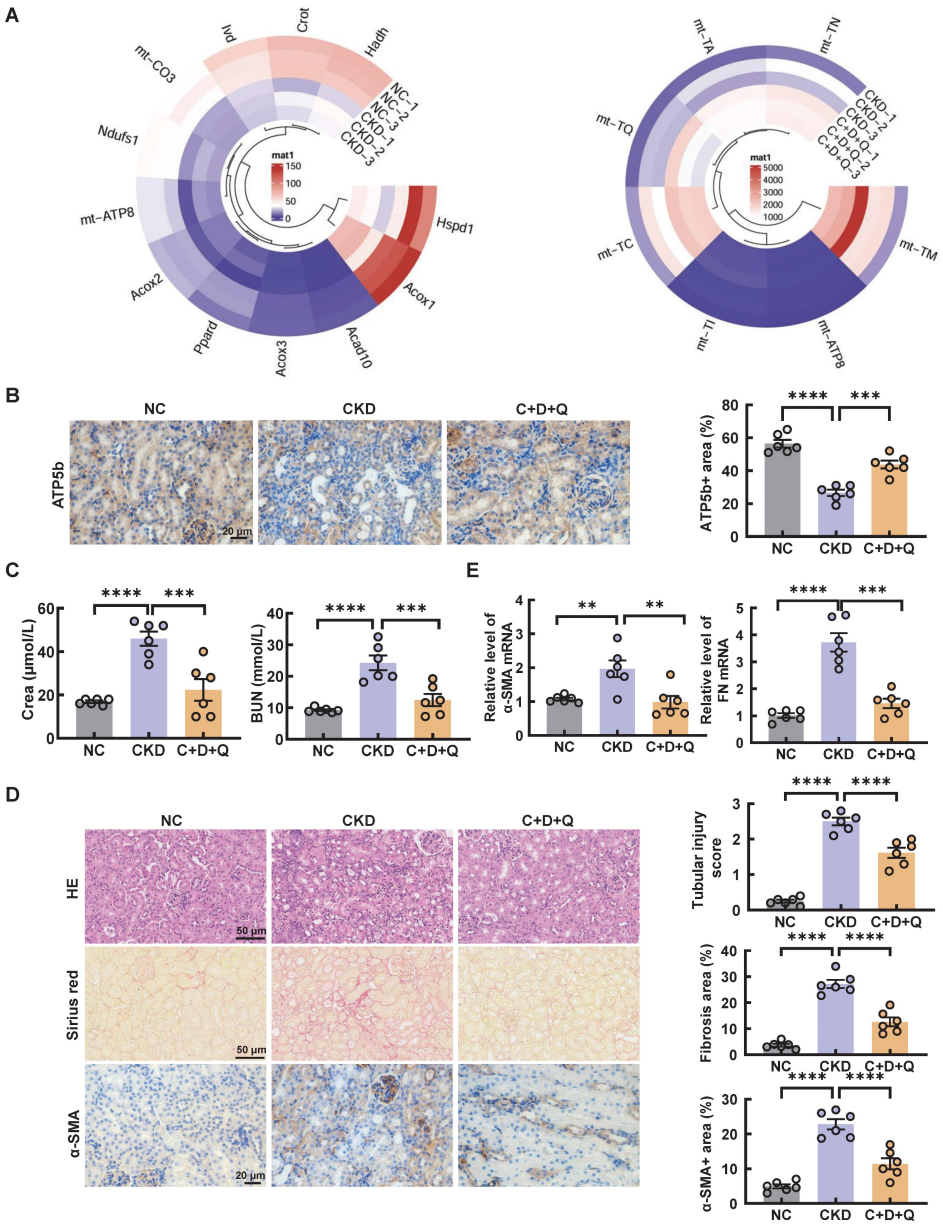
**D+Q treatment ameliorates renal injury in multiple cisplatin-induced CKD mice.** (A) Circular heatmap of mitochondrial-related genes in renal tissues associated with the 3 different groups. (B) Representative images of immunohistochemical staining of ATP5b. Scale bar, 20 μm. (C) The serum levels of CREA and BUN in the mice. (D) Representative images of hematoxylin-eosin (HE) and sirius red-staining (Sirius red), immunohistochemical staining and quantification of α-SMA in paraffin-embedded kidney sections. Scale bars, 50 µm and 20 µm. (E) The mRNA levels of α-SMA and fibronectin (FN) in the 3 different groups. n = 6 mice per group were used to analyze the results. Data are shown as the means ± SD from at least three independent experiments and analyzed by one-way ANOVA with Tukey's test. *P < 0.05, **P < 0.01, ***P < 0.001, ****P < 0.0001. (NC, normal control; CKD, cisplatin-induced CKD mice; C+D+Q, cisplatin-induced CKD mice were orally administered D plus Q).

Consistently, the senescence induced by cisplatin in CKD mice was significantly alleviated after the oral administration of D+Q (Figure 5C-D). Moreover, compared with that in the control group, the expression of mitochondrial-related genes, such as Ndufs1, Acox1, and mt-ATP8, was decreased in CKD mice; however, these changes were reversed by D+Q treatment, as shown by RNA-seq analysis (Figure 6A). In addition, the level of ATP5b increased after D+Q treatment (Figure 6B). Consequently, compared with CKD mice, D+Q administration reduced levels of Crea and BUN, mitigated pathological damage and renal interstitial fibrosis (Figure 6C-E). These findings suggest that D+Q treatment ameliorates renal injury in a murine model of multiple cisplatin treatments through a feedback loop between mitochondrial damage and cell senescence.

### Post-intervention of trehalose activates autophagy and ameliorates cisplatin-induced renal injury

**Figure 7:**
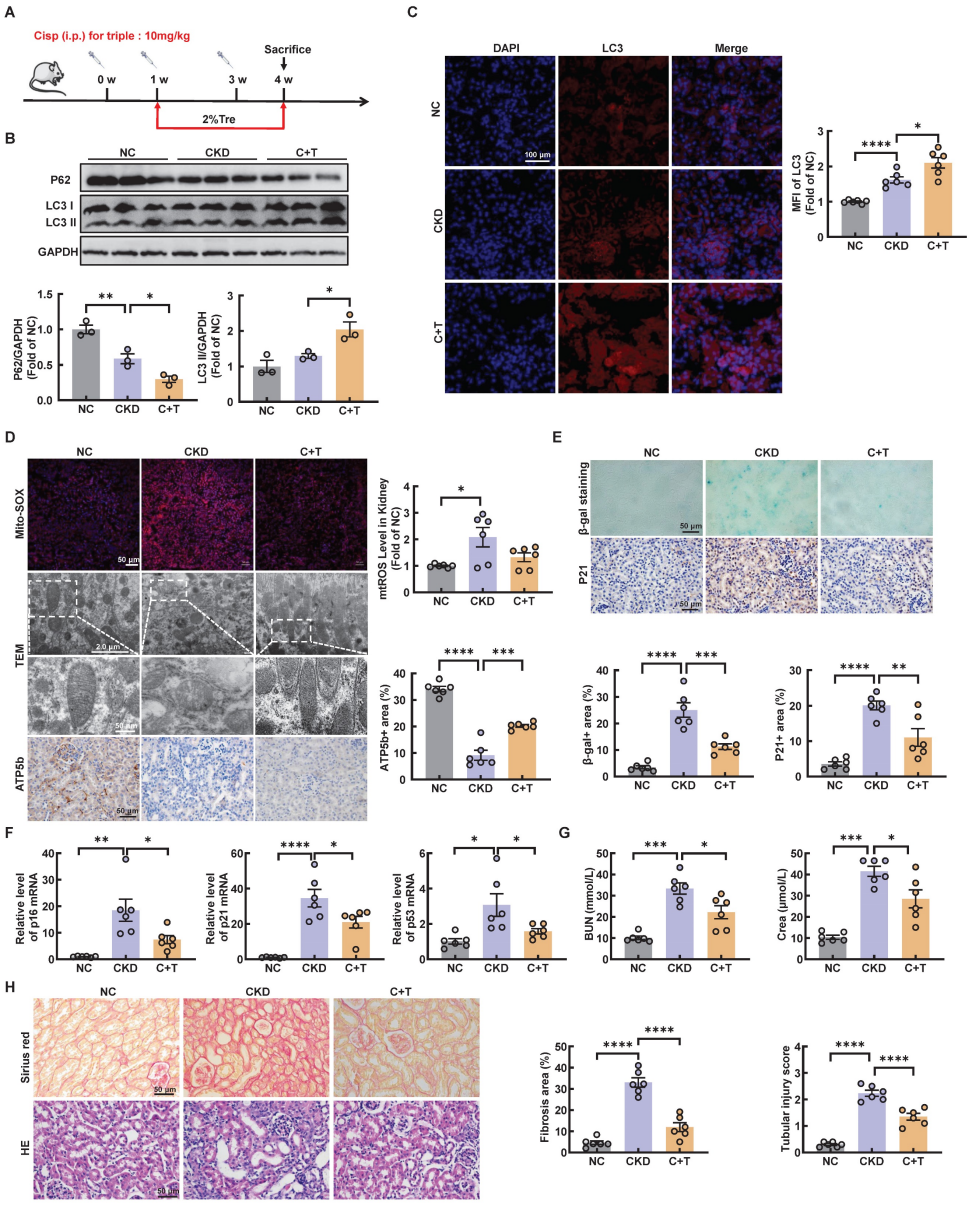
** Post-intervention of trehalose activates autophagy and ameliorates renal injury in CKD mice.** (A) Illustration of post-intervention of trehalose treatment in cisplatin-induced CKD mice. (B) Western blot and quantitative analyses of P62 and LC3 II. GAPDH was used as the loading control. (C) Representative immunofluorescence images and quantification of LC3 (red) in kidney sections. Scale bar, 100 μm. (D) Representative images showing mtROS in fresh renal tissue visualized by staining with mitoSOX. Scale bar, 50 μm. Representative TEM micrographs of mitochondria from each group. Scale bar, 2 μm and 50 nm. Images of ATP5b immunohistochemical staining. Scale bar, 50 μm. (E) Renal tissue was stained for SA-β-Gal activity. Images of immunohistochemical staining of P21. Scale bar, 50 μm. (F) Relative mRNA levels of p16, p21, and p53 were shown. (G) BUN and Crea levels were quantified in each group. (H) Representative images of sirius red-staining (Sirius red) and hematoxylin-eosin (HE) of kidney sections. n = 6 mice per group were used to analyze the results. Data are shown as the means ± SD from at least three independent experiments and analyzed by one-way ANOVA with Tukey's test. *P < 0.05, **P < 0.01, ***P < 0.001, ****P < 0.0001. (NC, normal control; CKD, cisplatin-induced CKD mice; C+T, cisplatin-induced CKD mice treated with 2% w/v trehalose solution to treat CKD mice one week after the first intraperitoneal injection of cisplatin).

We further investigated whether trehalose treatment attenuated kidney injury when renal function was impaired after cisplatin injection. The above results revealed that CKD mice treatment with 2% w/v trehalose solution had a better curative effect (Figure 1B-F). Accordingly, we used 2% w/v trehalose solution to treat CKD mice one week after the first intraperitoneal injection of cisplatin (Figure 7A). Consistent with previous trehalose treatment, post-intervention of trehalose also increased LC3II, while the level of P62 decreased, indicating that autophagy was activated after trehalose treatment (Figure 7B-C and Figure S6A). In addition to autophagy, mitochondrial dysfunction was inhibited after post-intervention of trehalose treatment (Figure 7D and Figure S6B). Moreover, cellular senescence was also suppressed, as indicated by immunohistochemistry, β-gal staining and RT-PCR (Figure 7E-F and Figure S6C). Finally, post-intervention of trehalose treatment, the levels of BUN and Crea were decreased, accompanied by improvements in pathological structural damage (Figure 7G-H). Collectively, these data also suggest that even post-intervention of trehalose also activates autophagy and reduces renal injury caused by cisplatin.

### Trehalose accumulates in lysosomes and inhibits lysosomal mTORC1 signaling

**Figure 8:**
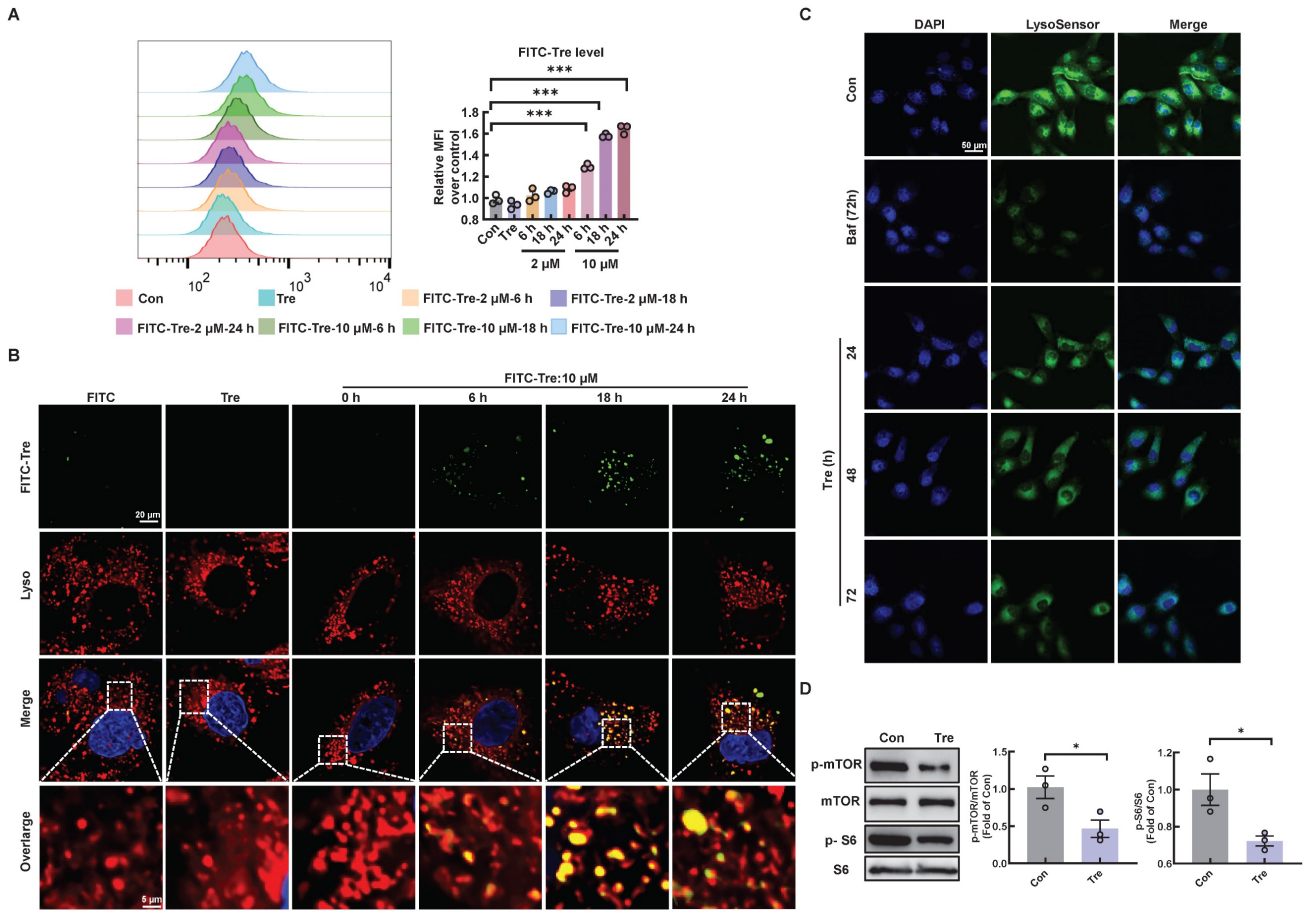
** Trehalose accumulates in lysosomes and inhibits lysosomal mTORC1 signaling.** (A) HK2 cells treated with FITC-conjugated trehalose at different durations and doses were detected by flow cytometry. (B) HK2 cells treated with FITC-conjugated trehalose were stained with the lysosomal marker and imaged by fluorescence microscopy for FITC (green) and LysoTracker Red (red), Scale bar, 20 μm and 5 μm. (C) HK2 cells incubated with trehalose or bafilomycin A1(10 nM) for 72h after staining with LysoSensor Green to monitor lysosomal acidity. (D) Western blot and quantitative analyses of p-mTOR/mTOR and p-S6/S6. Data are shown as the means ± SD from at least three independent experiments and analyzed by one-way ANOVA with Tukey's test and Student's t test. *P < 0.05, ***P < 0.001. (Con, control; Tre, trehalose; Baf, HK2 cells incubation with bafilomycin A1).

To elucidate the potential mechanism by which trehalose regulates autophagy, we first confirmed the intracellular uptake of trehalose via FACS analysis in HK2 cells incubated with trehalose conjugated to a FITC-conjugated fluorochrome. Here, trehalose intake was observed in a time- and concentration-dependent manner (Figure 8A). Then, co-staining of FITC-conjugated trehalose and the lysosomes confirmed that trehalose was taken up in HK2 cells and colocalization of with lysosomes (Figure 8B). Emerging evidence reports that the rise in intra-lysosomal trehalose concentration results in lysosomal stress including modest increase in lysosomal pH, which induces inactivation of mTOR 13, 24. Therefore, we first evaluated the effects of trehalose on lysosomal acidification. In this regard, we conducted HK2 cells treated with trehalose or Baf, a well-known lysosomotropic drug that inhibits lysosomal acidification. As illustrated, the rise in intra-lysosomal trehalose concentration results in modest increases in lysosomal pH, while the effects of Baf resulted in a disruption of lysosomal pH (Figure 8C). Then, we found that trehalose treatment decreased the expression of phosphorylation of mTOR and S6 (Figure 8D). Together, these data indicate that trehalose accumulates in lysosomes and inhibits lysosomal mTORC1 signaling.

### Trehalose triggers TFEB nuclear translocation via hampering the formation of the RagGTPasese-Ragulator lysosomal scaffold

**Figure 9:**
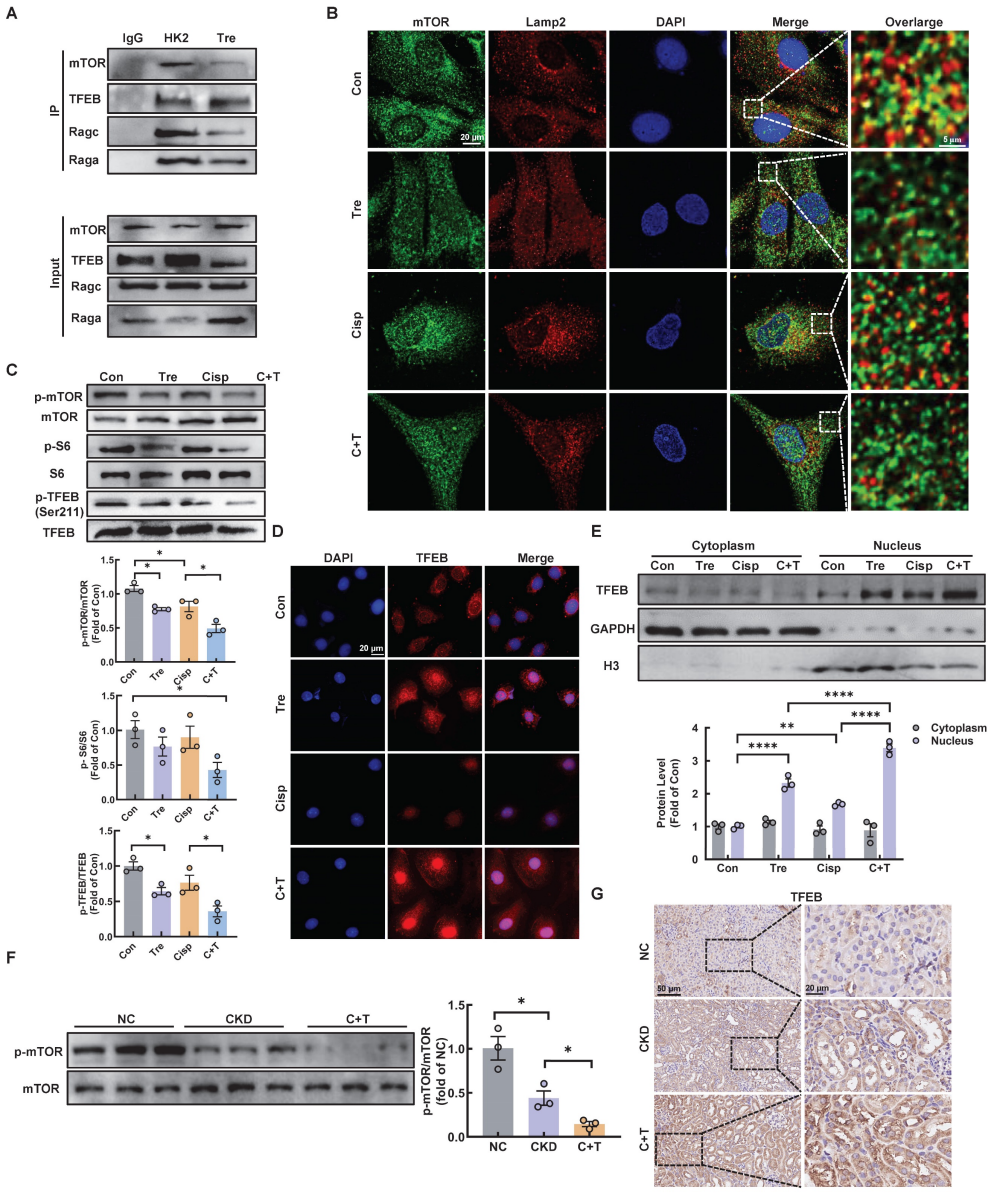
** Trehalose triggers TFEB nuclear translocation via hampering the formation of the RagGTPasese-Ragulator lysosomal scaffold.** (A) Representative co-immunoprecipitation (Co-IP) analysis to assay interactions between TFEB and RagA, RagC, mTOR in HK2 cells. (B) Representative immunofluorescence images of mTOR (green) and Lamp2 (red) in HK2 cells subjected to different stimuli. (C) Western blot and quantitative analyses of p-mTOR/mTOR, p-S6/S6 and p-TFEB (Ser 211)/TFEB. (D) Representative immunofluorescence images of TFEB (red) in HK2 cells. (E) Cytoplasmic and nuclear fractions of TFEB in HK2 cells were analyzed by western blotting (cisplatin, 20 μM; trehalose, 100 mM). (F) Western blot and quantitative analyses of p-mTOR/mTOR. (G) Representative images of immunohistochemical staining of TFEB in paraffin-embedded kidney sections. Scale bar, 50 μm and 20 μm. n = 6 mice per group were used to analyze the results. Data are shown as the means ± SD from at least three independent experiments and analyzed by one-way ANOVA with Tukey's test. *P < 0.05, **P < 0.01, ****P < 0.0001. (Con, control; Tre, trehalose; Cisp, cisplatin; C + T, cisplatin + trehalose; NC, normal control; CKD, cisplatin-induced CKD mice; C+T, Cisplatin-induced CKD mice were treated with 2% w/v trehalose solution to intervene in CKD mice one week after the first intraperitoneal injection of cisplatin).

Early studies demonstrated that mTORC1 is recruited to the lysosomal membrane and activated by Rag GTPase, which is a heterodimer composed of Rag A or B combined with Rag C or D 25. Rag GTPase-dependent lysosomal recruitment of mTORC1 is essential for TFEB phosphorylation by mTORC1 26, 27. Upon starvation stress, the dephosphorylation of TFEB translocates to the nucleus, resulting in activated transcription of autophagy-lysosomal related genes and promoting the autophagy 28. As illustrated, we found that trehalose administration decreased the interaction of mTOR with both RagA and RagC, as well as its interaction with TFEB, which induced the dissociation of mTOR from lysosome (Figure 9A-B). In addition, we evaluated the activity of MHY1485, an mTOR activator, in trehalose-treated HK2 cells. The increased nuclear translocation of TFEB in the presence of trehalose was suppressed by treatment with MHY1485 (Figure S7A). Finally, trehalose treatment suppressed mTOR signaling pathway (Figure 9C and 9F), and trigged TFEB nuclear localization *in vitro* and* in vivo* (Figure 9D-E and Figure 9G). Taken together, these results confirm that trehalose dephosphorylates TFEB and promotes its nuclear localization by hampering the assembly of the RagseRagulator scaffold at the lysosome and inhibiting mTORC1 activity.

### Trehalose induces autophagy by activating TFEB in cisplatin-treated models

**Figure 10:**
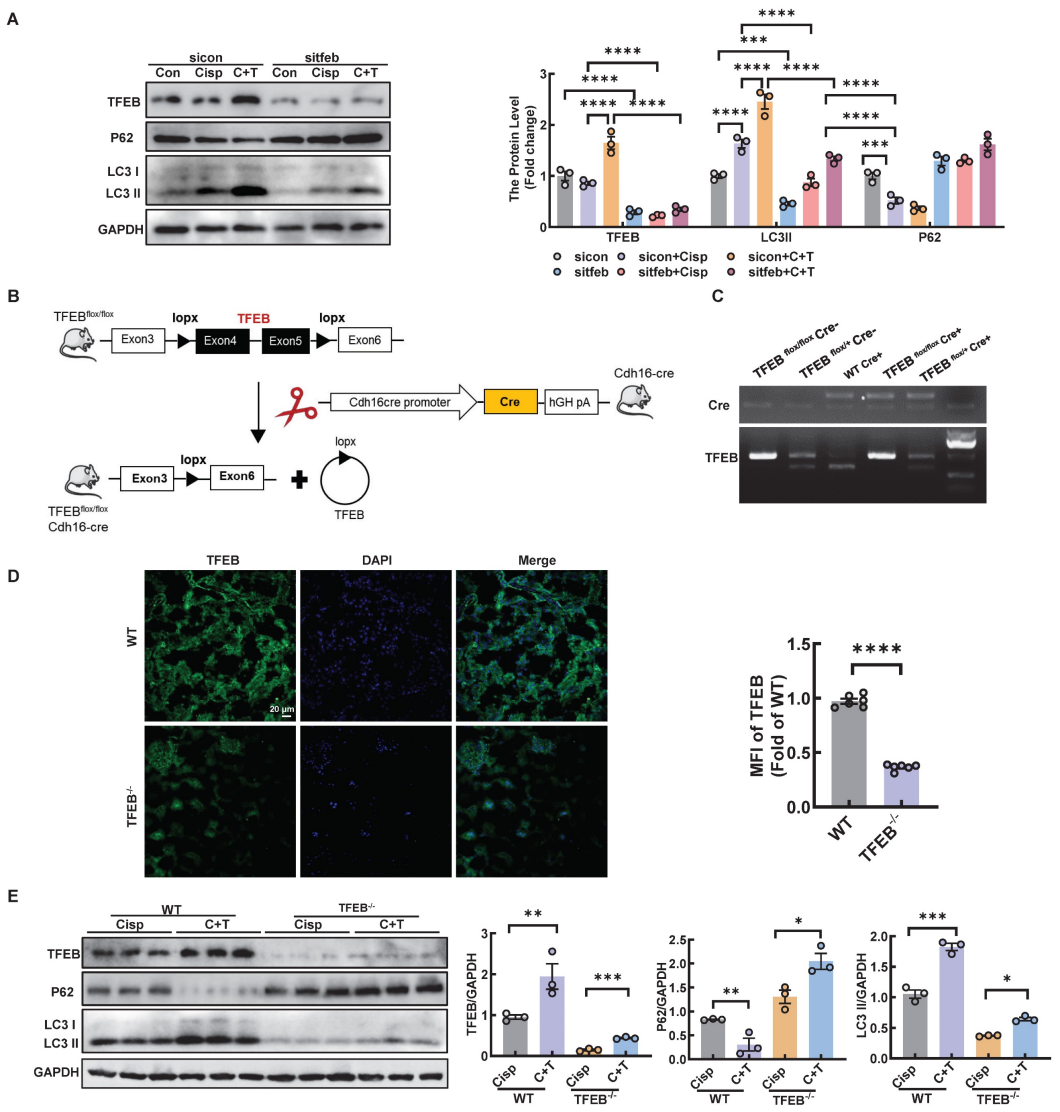
** Trehalose induces autophagy by activating TFEB in cisplatin-treated models.** HK2 cells were transfected with control siRNA (sicon) or TFEB siRNA (sitfeb) for 6 h and treated with cisplatin (20 μM) in the presence or absence of trehalose (100 mM) for 72 h. (A) Western blot and quantitative analyses of TFEB, P62 and LC3 II. GAPDH was used as the loading control. (B) Schematic diagram of the generation of a conditional knockout mouse by mating TFEB floxed mice expressing Cre recombinase under the renal proximal tubule cadherin promoter (Cdh16-Cre). (C) Genotype identification of TFEB ^flox/flox^ Cre^-^, TFEB ^flox/+^ Cre^-^, WT Cre^+^, TFEB ^flox/flox^ Cre^+^ and TFEB^ flox/+^ Cre^+^ mice. (D) Representative immunofluorescence images and quantification of TFEB (green) in kidney sections. Scale bar, 20 μm. (E) Western blot and quantitative analyses of TFEB, P62 and LC3 II. GAPDH was used as the loading control. n = 6 mice per group were used to analyze the results. Data are shown as the means ± SD from at least three independent experiments and analyzed by one-way ANOVA with Tukey's test and Student's t test. *P < 0.05, **P < 0.01, ***P < 0.001, ****P < 0.0001. (Con, control; Cisp, cisplatin; C + T, cisplatin + trehalose. WT, wild type; TFEB^-/-^, renal proximal tubule-specific TFEB deficient mice; Cisp, cisplatin-induced CKD mice; C+T, cisplatin-induced CKD mice treated with trehalose diluted in drinking distilled water at a final concentration of 2% w/v solution).

To determine whether the activation of trehalose on autophagy was relies on TFEB, HK2 cells were transfected with sitfeb *in vitro* (Figure 10A). Silencing of TFEB decreased the level of LC3 II, whereas the expression of p62 increased, suggesting that the autophagy process was inhibited in the cisplatin-treated models (Figure 10A). *In vivo*, we used Cre-loxP system to knock out TFEB in proximal tubular epithelial cells (Figure 10B-D). The level of LC3 II was significantly reduced, while the level of P62 was elevated (Figure 10E). Taken together, these results indicate that TFEB is crucial for the induction of autophagy upon trehalose treatment.

### Silencing TFEB partially abolishes the protective effects of trehalose *in vitro* and* in vivo*

**Figure 11:**
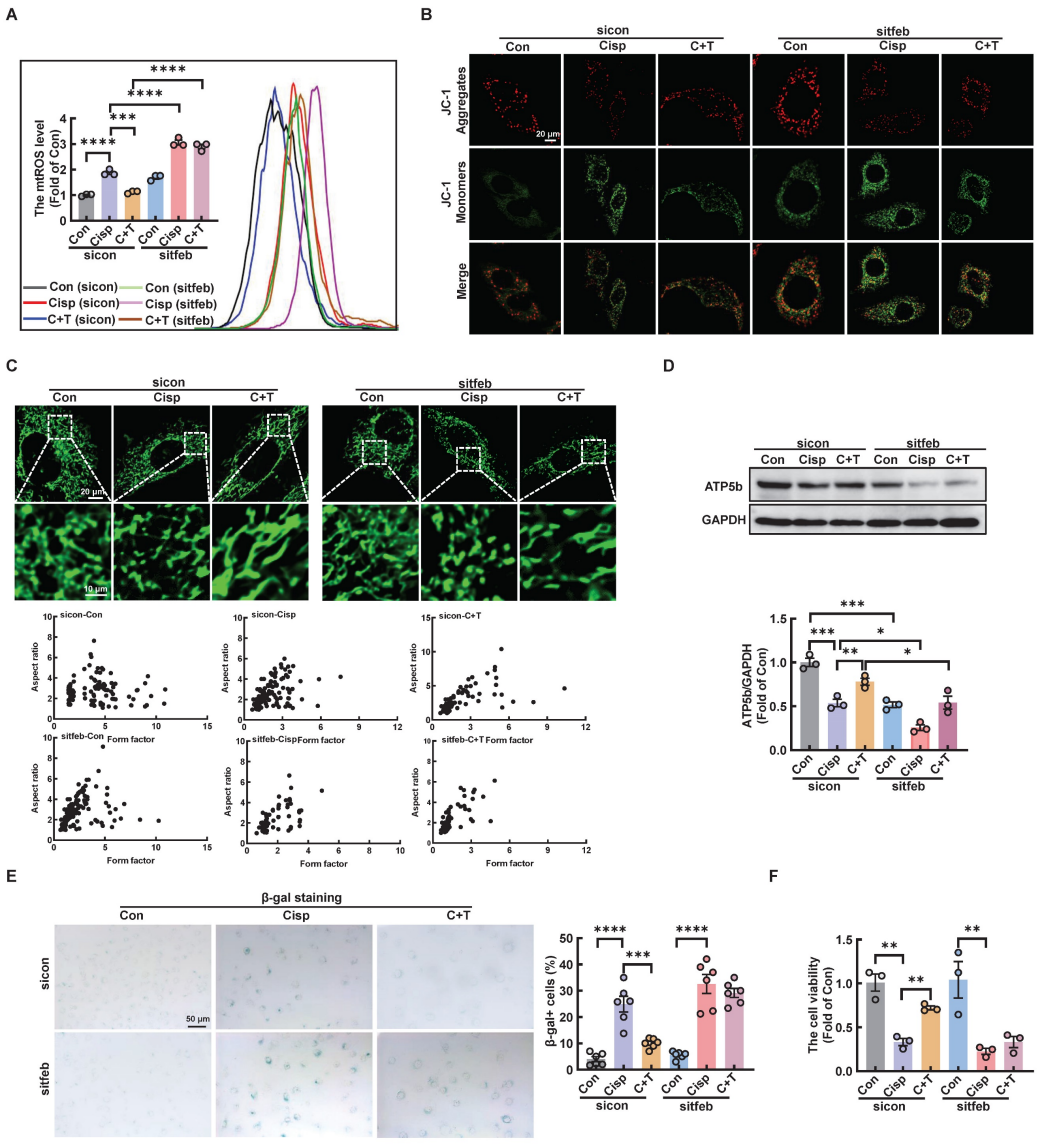
** Silencing TFEB partially abolishes the protective effects of trehalose in cisplatin-treated HK2 cells.** (A) mtROS were measured by flow cytometry following incubation with MitoSOX. (B) Detection of JC-1 aggregates (red) and monomers (green) in HK2 cells by confocal fluorescence microscopy. (C) Representative images of MitoTracker Green fluorescence in HK2 cells subjected to different treatments. (D) Western blot and quantitative analyses of ATP5b. GAPDH was used as the loading control. (E) HK2 cells were stained for SA-β-Gal. (F) TFEB-knockdown HK2 cells were exposed to cisplatin (20 μM) for 6 h and observed in complete medium for 72 h, and cell viability was determined by CCK8. Data are shown as the means ± SD from at least three independent experiments and analyzed by one-way ANOVA with Tukey's test. *P < 0.05, **P < 0.01, ***P < 0.001, ****P < 0.0001. (Con, control; Cisp, cisplatin; C + T, cisplatin + trehalose).

We further explored whether the protective role of trehalose in cisplatin-induced HK2 cells was dependent on TFEB-mediated autophagy. Compared with trehalose treatment, when the TFEB was knocked out in HK-2 cells, the increased cell viability, inhibition of mitochondrial dysfunction and senescence were remarkedly abrogated (Figure 11A-F and Figure S8A).

**Figure 12:**
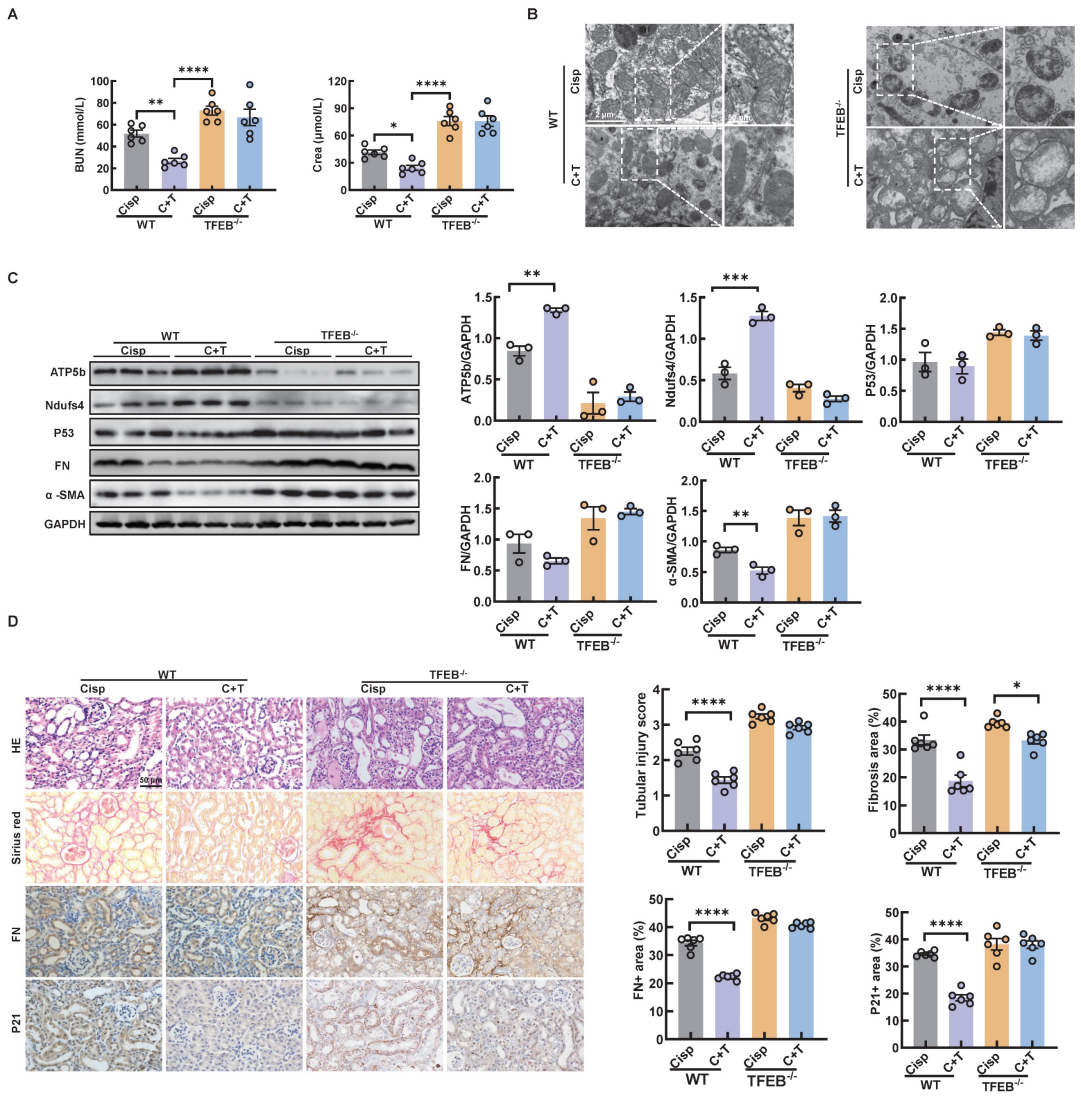
** TECs-specific deletion of TFEB abolishes the protective effects of trehalose in CKD mice.** (A) The serum levels of BUN and CREA in the mice. (B) Representative TEM micrographs of mitochondria in kidney sections from each group. Scale bar, 2 μm and 50 nm. (C) Western blot and quantitative analyses of ATP5b, Ndufs4, P53, fibronectin (FN), and α-SMA. GAPDH was used as the loading control. (D) Representative images of hematoxylin-eosin (HE) and sirius red-staining (Sirius red), immunohistochemical staining and quantification of fibronectin (FN) and P21 in paraffin-embedded kidney sections. Scale bars, 50 µm. n = 6 mice per group were used to analyze the results. Data are shown as the means ± SD from at least three independent experiments and analyzed by one-way ANOVA with Tukey's test. *P < 0.05, **P < 0.01, ***P < 0.001, ****P < 0.0001. (WT, wild type; TFEB^-/-^, renal proximal tubule-specific TFEB mice; Cisp, cisplatin-induced CKD mice; C+T, cisplatin-induced CKD mice were treated with trehalose diluted in drinking distilled water at a final concentration of 2% w/v solution).

In an effort to elucidate the underlying mechanism by which autophagy alleviates CKD in mice, we measured the renal function in TECs-specific deletion of TFEB^-/-^ mice. First, we found that the TECs-specific deletion of TFEB with or without the treatment of trehalose for 4 weeks had no effect on mouse blood BUN and Crea levels (Figure S9A). In addition, no pathological changes in the structure of the kidney were observed, suggesting that TECs-specific deletion of TFEB under physiological condition has no effect on kidney (Figure S9B). Whereas, under multiple injection of cisplatin-induced CKD state, TECs-specific deletion of TFEB markedly abrogated the protective effect of treatment, as evidenced by serum biochemistry, mitochondrial dysfunction and senescence (Figure 12A-D). Together, these results may suggest that TFEB-mediated autophagy is critical to the protective effects of trehalose in cisplatin-induced injury models.

## Discussion

Our previous observations indicated that trehalose treatment activated TFEB-mediated autophagy and attenuated mitochondrial dysfunction and kidney injury in cisplatin-induced AKI mice 14. However, reports on the role of autophagy in CKD are contradictory and inconsistent. Our aim is to explore the effect of trehalose-activated autophagy on CKD intervention and to explore the molecular mechanism of the mTOR/TFEB pathway in this process by using renal proximal tubule cells-specific deletion of TFEB mice for the first time.

In order to evaluate the safety of trehalose treatment and its potential impact on the efficacy of cisplatin-induced CKD. Normal control mice were administrated with trehalose via two different ways for 4 weeks, no adverse effects were observed, as indicated by the levels of blood urea nitrogen, serum creatinine and pathological section (Figure S4). In addition, we also used the well-known nephroprotective agent resveratrol (RSV) to compare its effect with trehalose. As illustrated, both trehalose and resveratrol relieved cisplatin-induced CKD kidney injury (Figure S10A-B). However, Liu *et al.* reported that higher doses of RSV promoted kidney fibrosis in the UUO mice 29. Zaltzman *et al.* demonstrated that 30 g 10% trehalose solution could be well tolerated, and no serious drug-related adverse events were observed over a period of up to 12 months in Machado-Joseph disease (MJD) patients 30. In addition, in an open study of 25 oculopharyngeal muscular dystrophy (OPMD) patients who received trehalose for 24 weeks, no serious drug-related adverse effects were noted 31. Thus, these studies showed the safety and efficacy of trehalose, suggesting it might be therapeutic potential in CKD.

Emerging evidence has revealed that trehalose potentiates autophagy by activating TFEB 14, 32, 33. Our study has found that trehalose could activate TFEB-mediated autophagy to suppress mitochondrial damage and senescence. Aa s consequence, the kidney injury in cisplatin-induced CKD mice was alleviated. The activity of TFEB is controlled by posttranslational modifications and protein‒protein interactions 34. Phosphorylated TFEB primarily resides in the cytoplasm, while its dephosphorylated form translocates to the nucleus to regulate autophagy-related gene expression. At least 10 phosphorylation sites have been identified, indicating complex regulation. Key phosphorylation events include Ser467 by AKT, Ser142 by ERK2 and Ser142/Ser211 by mTORC1, both of which critically influence TFEB's subcellular localization 35, 36. Here, we observed that trehalose significantly decreased the expression of p-mTOR, whereas treatment with trehalose did not affect the expression of p-ERK and p-AKT* in vitro* (Figure 9C and Figure S11A).

Data from preclinical studies suggest that activation of mTORC1 has been detected in kidney tissue from patients with diabetic kidney disease (DKD), focal segmental glomerulosclerosis (FSGS) and Polycystic kidney disease (PKD) 37, 38. Moreover, the similar phenomenon also been observed in unilateral ureteral obstruction (UUO) and folic acid (FA)-induced CKD model 37. Nevertheless, in our study, we found that the expression of p-mTOR and p-S6 was decreased at 4th week, while gradually increased at 6-8 weeks (Figure S12A-B), indicating that the activity of mTOR was inhibited firstly and then elevated along with the process of cisplatin-induced CKD. Jeong *et al.* have proved that “low-grade” lysosomal stress can inhibit the mTOR activity. Similar with the observation, we found the number of damaged lysosome (Gal3+) in kidney at 4th week was increased (Figure S12C). However, the lysosomal degradative capacity in the autophagy process was not affected, demonstrated by the increased expression of LC3 II and the reduction of P62 (Figure S2B). Therefore, we speculated that the inactivation of mTOR at the early stage was due to the “low-grade” lysosomal stress. Additionally, trehalose administration could further inhibit mTOR activity, and suppress the phosphorylation of TFEB, leading to the induction of autophagy* in vivo*. More importantly, to verify whether autophagy relies on TFEB, we further knocked out TFEB in HK2 cells and tubular epithelial cells in mice, and the results showed that the improvement induced by trehalose treatment was remarkably abrogated by TFEB knockdown in HK2 cells and mice, indicating that the protective role of trehalose in CKD relied on the mTOR-TFEB axis.

Cell membranes are known to be impermeable to disaccharides including trehalose, suggesting it must either interact with receptors on the plasma membrane or be internalized to exert intracellular effects. Interestingly, our research revealed that trehalose could be endocytically taken up by cells and accumulated within the endolysosomal system, which caused a mild elevation of lysosomal pH, and subsequently impaired the interaction of the Ragulator complex with Rag GTPases, thereby blocking lysosomal localization and activity of mTORC1.

Given that trehalose activated TFEB-mediated autophagy and alleviated kidney injury in cisplatin-induced CKD mice, much more CKD models, such as UUO, diabetic nephropathy, or indoxyl sulfate-induced CKD should be applied to systematically evaluate the therapeutic effects of trehalose. Additionally, due to the influence of gender on drug metabolism and disease progression, studies evaluating the ability of trehalose to ameliorate CKD in female mice will be required to fully evaluate its optimal role.

## Conclusions

In summary, we demonstrated that trehalose could be endocytically taken up by cells and accumulated within lysosome, which inactivated of mTOR and subsequently promoted TFEB-mediated autophagy, leading to the alleviation of cisplatin-induced CKD. These findings not only increase our understanding of the pathogenesis of CKD but also provide therapeutic potential by activation mTOR-TFEB-autophagy axis.

## Materials and Methods

### Materials and reagents

Cisplatin and hydroxychloroquine (HCQ) were obtained from MedChemExpress (MCE, New Jersey, USA). Trehalose was obtained from ApexBio (Boston, MA, USA). Dasatinib (D) was obtained from Selleck. Quercetin (Q) and bafilomycin A1 were obtained from Sigma‒Aldrich. The Senescence β-Galactosidase Staining Kit was obtained from Beyotime. The cell counting kit 8 (CCK8) assay was from Dojindo (Kumamoto, Japan). MitoSOX Red, MitoTracker Deep Red and Hoechst were obtained from Thermo Fisher Scientific (Sunnyvale, CA, USA). A mitochondrial membrane potential (MMP) assay kit (JC-1) was obtained from AAT Bioquest (Sunnyvale, CA). MitoTracker Green was obtained from Beyotime Biotechnology (Jiangsu, China). TFEB-siRNA was synthesized by GenePharma (Shanghai, China). JetPRIME transfection reagent was obtained from Polyplus Transfection (Illkirch, France). The reagents used for transmission electron microscopy (osmium tetroxide, Epon, and lead citrate) and DAPI were obtained from Sigma‒Aldrich (Taufkirchen, Germany). The following primary antibodies were used: anti-LC3B, anti-TFEB (Cell Signaling Technology, Beverly, MA); anti-Ndufs4, anti-α-SMA, anti-ATP5b, anti-P53, anti-GAPDH and anti-Fibronectin (ABclonal Biotech Co., Ltd., Cambridge, MA, USA), anti-P62 (Abcam), anti- RagA, anti-RagC, anti-p-S6, anti-S6, anti-mTOR and anti-p-mTOR (Huabio). For real-time PCR, TRIzol was obtained from Gibco (Life Technologies, CA, USA), the SYBR Green PCR mix was obtained from Vazyme Biotech (Nanjing, China), and the iScript cDNA synthesis kit was obtained from Bio-Rad (CA, USA).

### Cell line and culture conditions

The human renal proximal tubular cell line (HK2) was obtained from the American Type Culture Collection (ATCC) and used for the experiments. The cells were grown in DMEM/F-12 (HyClone, Solarbio, Beijing, China) supplemented with 10% fetal bovine serum, 1% penicillin-streptomycin at 37 °C in 5% CO_2_ in a humidified incubator.

Cells were incubated with 20 μM cisplatin for 6 h, followed by treatment with medium and observation at 24 h, 48 h, and 72 h 4. To detect autophagy, 100 mM trehalose or 20 μM HCQ was added to cells treated with cisplatin.

### RNA interference

Cells were transfected with small interfering RNA specific for the TFEB gene (sitfeb) (sense: GACGAAGGUUCAACAUCAATT; antisense: UUGA UGUUGAACCUUCGUCTT) or negative control RNAi (sicon) from GenePharma (Shanghai, China). Cells were transfected with sitagliptin using jetPRIME transfection reagent.

### Mitochondrial membrane potential and mtROS determination

For mitochondrial membrane potential detection, cells were stained with tetrechloro-tetraethylbenzimidazol carbocyanine iodide (JC-1, 10 μg/ml; Beyotime, China) at 37 ℃ for 20 min. For analysis of mitochondrial superoxide, cells were incubated with 4 μM MitoSOX Red superoxide indicator (Invitrogen, USA) at 37 ℃ for 30 min, and mtROS quantification was performed using flow cytometry and visualization under a confocal microscope.

### Animals and treatment

Our study exclusively examined male mice. Male C57BL/6 mice (8 weeks old, weight 27-30 g) were originally obtained from Dashuo Biotechnology (Chengdu, China) and bred in the animal center of West China Hospital, Sichuan University, in accordance with the Guide for the Care and Use of Laboratory Animals. Mice were administered 10 mg/kg doses of cisplatin by three intra-peritoneal injections. We set the timing of the injections at 0, 1, and 3 weeks. All the recipients were sacrificed at 4 weeks after the first cisplatin injection. To investigate the effects of trehalose on cisplatin-induced CKD, trehalose was orally administered diluted in drinking distilled water at a final concentration of 2% w/v solution or at 1 g/kg of body weight via intraperitoneal injection, and 48 hours later, 2% w/v solution was administered to the treatment group. To assess the role of mitochondrial dysfunction in senescence, dasatinib (5 mg/kg) plus quercetin (50 mg/kg) (dasatinib + quercetin, D+Q) was delivered by oral gavage once a week. Mice were killed at 4 weeks after the first cisplatin injection. After euthanization, the blood and kidneys were collected for experiments.

TFEB^flox/flox^ mice (obtained from Shanghai Model Organisms Center, Inc., Cat. NO. NM-CKO-210046) and Cdh16-Cre mice (obtained from Cyagen Biosciences Inc., Suzhou, China, Cat. NO. C001452) were crossed to obtain renal proximal tubular epithelial cells -specific TFEB deficient mice.

All animal experiments were approved by the Animal Care and Use Committee of West China Hospital, Sichuan University (NO.20220224083) and were conducted according to the National Institutes of Health Guide for the Care and Use of Laboratory Animals.

### SA-β-gal staining

Tubular senescence changes were determined by SA-β-gal staining using a senescence detection kit (Beyotime, China) following the manufacturer's protocols.

### RNA isolation and RT-PCR

Total RNA was extracted from cultured cells or snap-frozen kidney tissues using TRIzol (Life Technologies, USA) and was converted to single-stranded cDNA with an iScript cDNA synthesis kit following the manufacturer's protocol. RT-PCR was performed using SYBR Green MasterMix (Vazyme, China) in a PCR detector (Bio-Rad). Glyceraldehyde-3-phosphate dehydrogenase (GAPDH) was used as a reference gene. The sequences of primers used for RT-PCR are listed in Table 1.

### Western blotting

Cultured cells or kidney lysates (renal parenchyma, including cortex and medulla) were extracted in RIPA buffer supplemented with protease inhibitor. The protein concentration was determined using a bicinchoninic acid (BCA) assay kit. Thirty micrograms of protein were loaded onto gels and separated by SDS‒PAGE, after which the proteins were transferred to a PVDF membrane. Immunoblotting was performed using a primary antibody overnight at 4 ℃. The following antibodies were used: anti-LC3B, anti-TFEB (Cell Signaling Technology); anti-Ndufs4, anti-α-SMA, anti-ATP5b, anti-P53, anti-GAPDH and anti- Fibronectin (ABclonal Biotech Co., Ltd.); anti-P62 (Abcam); anti-mTOR and anti-p-mTOR (Huabio). Then, the membranes were incubated with the corresponding secondary antibodies for 1 to 2 hours at room temperature. The immunoblots were visualized using a ChemiDoc™ imaging system (Bio-Rad, USA). The immunoblot band density was quantified via densitometry using ImageJ software.

### Co-immunoprecipitation

The cells were lysed with RIPA buffer supplemented with protease and phosphatase inhibitors. The supernatants were incubated with anti-TFEB or anti-IgG antibodies overnight at 4 ℃. After 5 washes, immunoprecipitation proteins were fractionated by SDS-PAGE and analyzed by western blotting with the indicated primary antibodies.

### Serum biochemistry

Plasma samples were separated by centrifugation at 1000 × g for 15 min. Clinical biochemical analysis of creatinine (Crea) and urea nitrogen (BUN) in the mice was performed by the Department of Laboratory Medicine of West China Hospital (Chengdu, China).

### Histological assessment

The kidney tissues were fixed with 4% paraformaldehyde and embedded in paraffin, and 5 μm paraffin sections were mounted on slides. Briefly, after deparaffinization and dehydration, the sections were subjected to antigen retrieval and blocked with 1% BSA before they were incubated with primary antibodies against ATP5b (1:500 dilution, A5769; ABclonal), Fibronectin (1:1000, ABclonal), α-SMA (1:1000, ABclonal), P21 (1:1000, ABclonal), and P53 (1:1000, ABclonal) overnight at 4 °C. In addition, kidney sections were stained with hematoxylin-eosin (HE) and Sirius Red and observed by light microscopy (Zeiss, Germany).

### Transmission electron microscopy (TEM)

Kidney tissues were fixed in paraformaldehyde and glutaraldehyde, postfixed in osmium tetroxide, dehydrated in ethanol, subjected to resin penetration and embedded in Epon. After that, the tissues were cut into ultrathin sections, stained with uranyl acetate, stained with lead citrate, and examined with an FEI Tecnai Spirit (T12). The aspect ratio and mitochondrial area were calculated.

### Statistical analysis

The quantitative data are expressed as the means ± SD of at least three independent experiments. Statistical differences between two groups were determined using a 2-tailed, unpaired Student's t test. Statistical differences among multiple groups were compared using one-way tests with multiple comparisons. Statistical significance was interpreted for P values less than 0.05.

## Supplementary Material

Supplementary figures.

## Figures and Tables

**Table 1:** Primers used for RT-PCR analysis.

Gene	Sequence 5'-3'	Species
GAPDH	Forward: CAGGAGGCATTGCTGATGAT Reverse: GAAGGCTGGGGCTCATTT	Human
IL-1β	Forward: GCCAGTGAAATGATGGCTTATT Reverse: AGGAGCACTTCATCTGTTTAGG	Human
TGF-β	Forward: CTGTACATTGACTTCCGCAAG Reverse: TGTCCAGGCTCCAAATGTAG	Human
IL-6	Forward: CACTGGTCTTTTGGAGTTTGAG Reverse: GGACTTTTGTACTCATCTGCAC	Human
p16	Forward: CCGTGGACCTGGCTGAGGAG Reverse: CGGGGATGTCTGAGGGACCTTC	Huamn
p21	Forward: GCCCGTGAGCGATGGAACTTC Reverse: CCTGCCTCCTCCCAACTCATCC	Huamn
p53	Forward: GCCCATCCTCACCATCATCACAC Reverse: GCACAAACACGCACCTCAAAGC	Huamn
GAPDH	Forward: GGTTGTCTCCTGCGACTTCA Reverse: TGGTCCAGGGTTTCTTACTCC	Mouse
p16	Forward: TCAAGACATCGTGCGATATTTG Reverse: TTAGCTCTGCTCTTGGGATTG	Mouse
p21	Forward: ATGTCCAATCCTGGTGATGTC Reverse: GAAGTCAAAGTTCCACCGTTC	Mouse
p53	Forward: TGGAAGGAAATTTGTATCCCGA Reverse: GTGGATGGTGGTATACTCAGAG	Mouse
α-SMA	Forward: CGTGGCTATTCCTTCGTGACTACTG Reverse: CGTCAGGCAGTTCGTAGCTCTTC	Mouse
FN	Forward: CTATAGGATTGGAGACACGTGG Reverse: CTGAAGCACTTTGTAGAGCATG	Mouse

## Abbreviations

AKI: acute kidney injury
Baf: Bafilomycin A1
BUN: blood urea nitrogen
CCK8: cell counting kit 8
Cisp: cisplatin
CKD: chronic kidney disease
C + T: cisplatin + trehalose
Con: control
Crea: serum creatinine
D: Dasatinib
HCQ: hydroxychloroquine
C + H: cisplatin + hydroxychloroquine
LC3 II: microtubule-associated protein 1 light chain 3-II
NC: normal control
Q: Quercetin
Rapa: rapamycin
ROS: reactive oxygen species
RSV: resveratrol
SDS-PAGE: sodium dodecyl sulfate-polyacrylamide gel electrophoresis
TFEB: transcription factor EB
Tre: trehalose
