# Supplementary material for: Trehalose activates autophagy to alleviate cisplatin-induced chronic kidney injury by targeting the mTOR-dependent TFEB signaling pathway
Source: Theranostics. 2025 Jan 20;15(6):2544–63. doi: 10.7150/thno.102559 (PMC11840734; doi:10.7150/thno.102559)
Supplement: Supplementary file 1 — Supplementary figures. [file thnov15p2544s1.pdf]

Supplementary Material

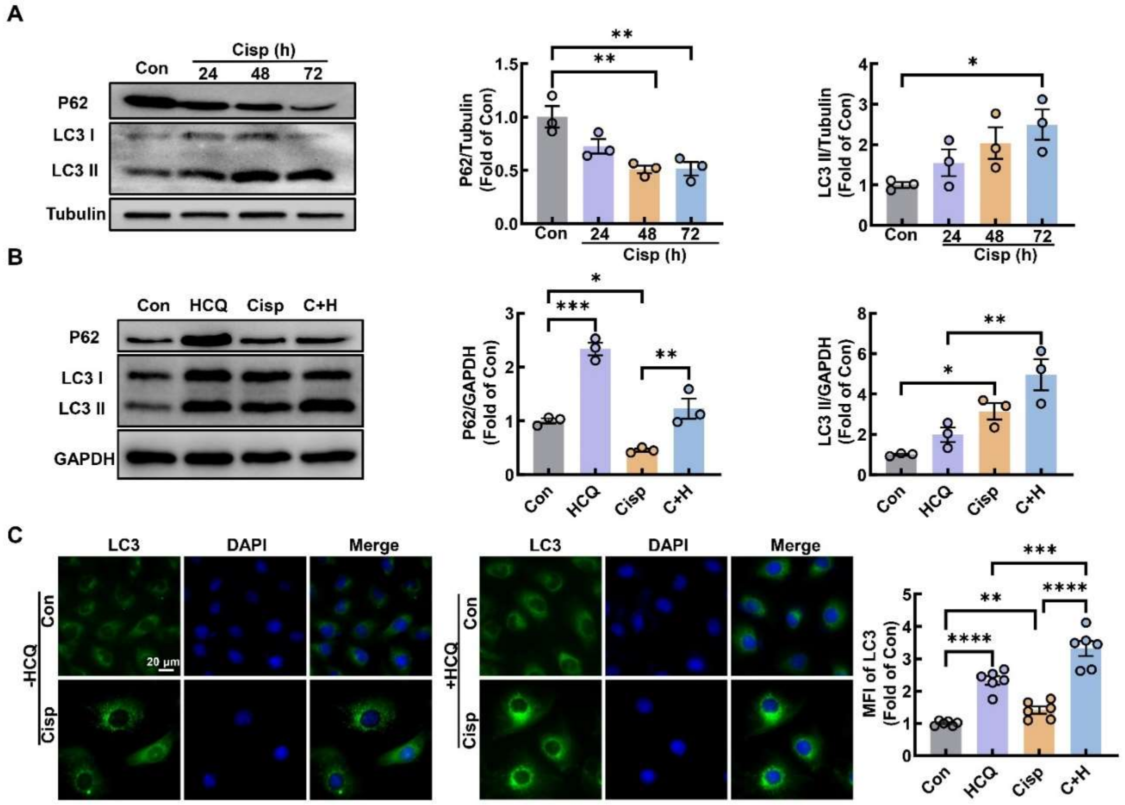

Figure S1 Autophagy is induced in cisplatin-treated HK2 cells. (A) HK2 cells were exposed to cisplatin (20 μM) for 6 h and then incubated and observed in complete medium for 24 h, 48 h, or 72 h. Western blot and quantitative analyses of P62 and LC3 II in kidney section. Tubulin was used as the loading control. (B) Western blot and quantitative analyses of P62 and LC3 II in HK2 cells exposed to cisplatin (20 μM) in the presence or absence of HCQ (20 μM). GAPDH was used as the loading control. (C) HK2 cells were treated with cisplatin (20 μM) in the presence or absence of HCQ (20 μM) for 6 h, and the expression of LC3 (green) was measured by immunofluorescence. Scale bar, 20 μm. Data are shown as the means ± SD from at least three independent experiments and analyzed by one-way ANOVA with Tukey's test. *P <0.05, **P <0.01, ***P <0.001, ****P <0.0001. (Con, control; Tre, trehalose; Cisp, cisplatin; C+T, cisplatin + trehalose, C+H, cisplatin + HCQ).

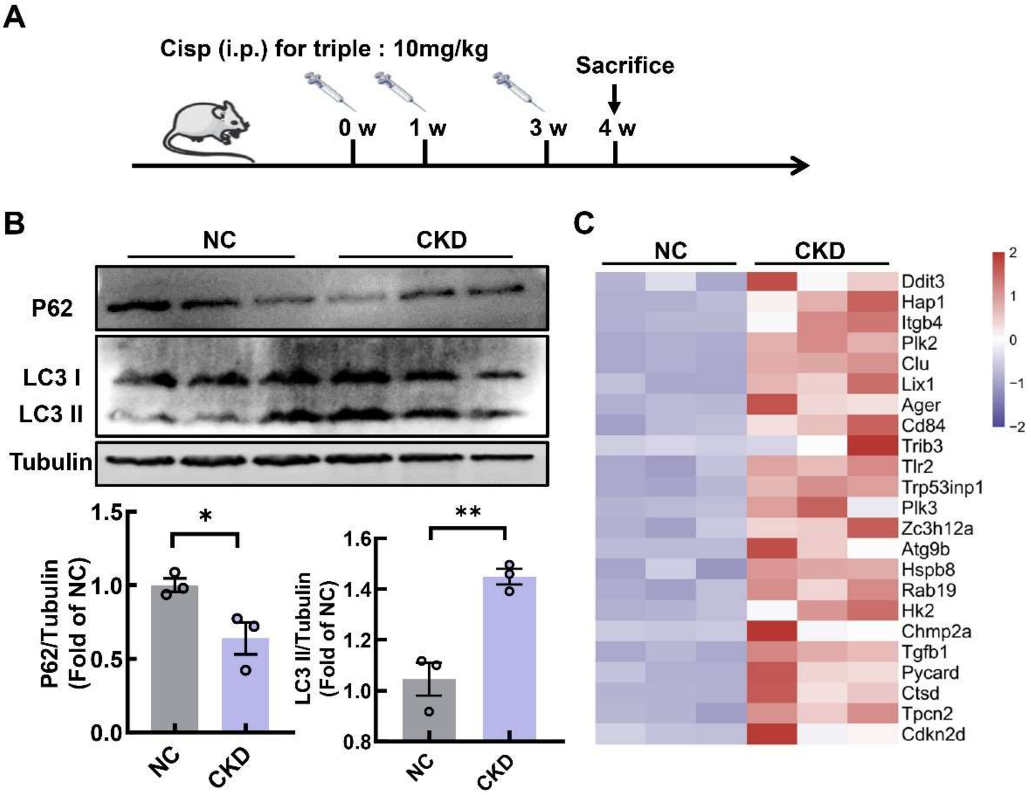

Figure S2 Autophagy is induced in cisplatin-treated CKD mice. (A) Illustration of cisplatin treatment in mice. Mice were intraperitoneally injected with three doses of cisplatin at a concentration of 10 mg/kg at 0, 1, and 3 weeks. (B) Western blot and quantitative analyses of P62 and LC3 II in kidney section. Tubulin was used as the loading control. (C) Heatmap representing the differential expression of autophagy-related genes in the NC group and cisplatin-induced CKD group. n = 6 mice per group were used to analyze the results. Data are shown as the means ± SD from three independent experiments and analyzed by Student's t test. *P <0.05, **P <0.01. (NC, normal control; CKD, cisplatin-induced CKD mice).

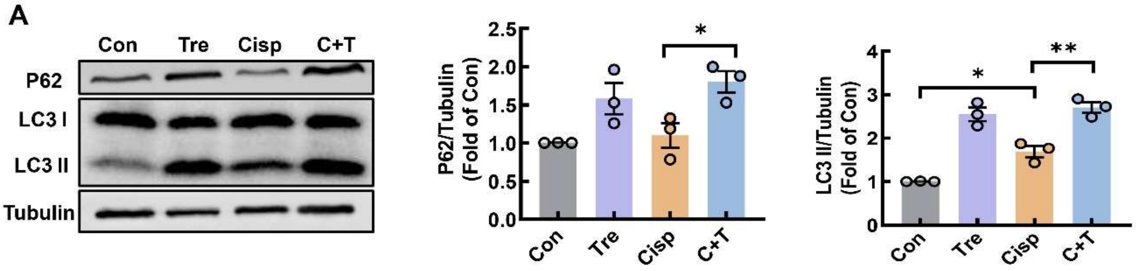

Figure S3 Tre further activates autophagy. (A) HK2 cells were treated with cisplatin (20 μM) in the presence or absence of trehalose (100 μM), and the levels of P62 and LC3 II were measured by western blotting. Data are shown as the means ± SD from three independent experiments and analyzed by one-way ANOVA with Tukey's test. *P <0.05, **P <0.01. (Con, control; Tre, trehalose; Cisp, cisplatin; C + T, cisplatin + trehalose).

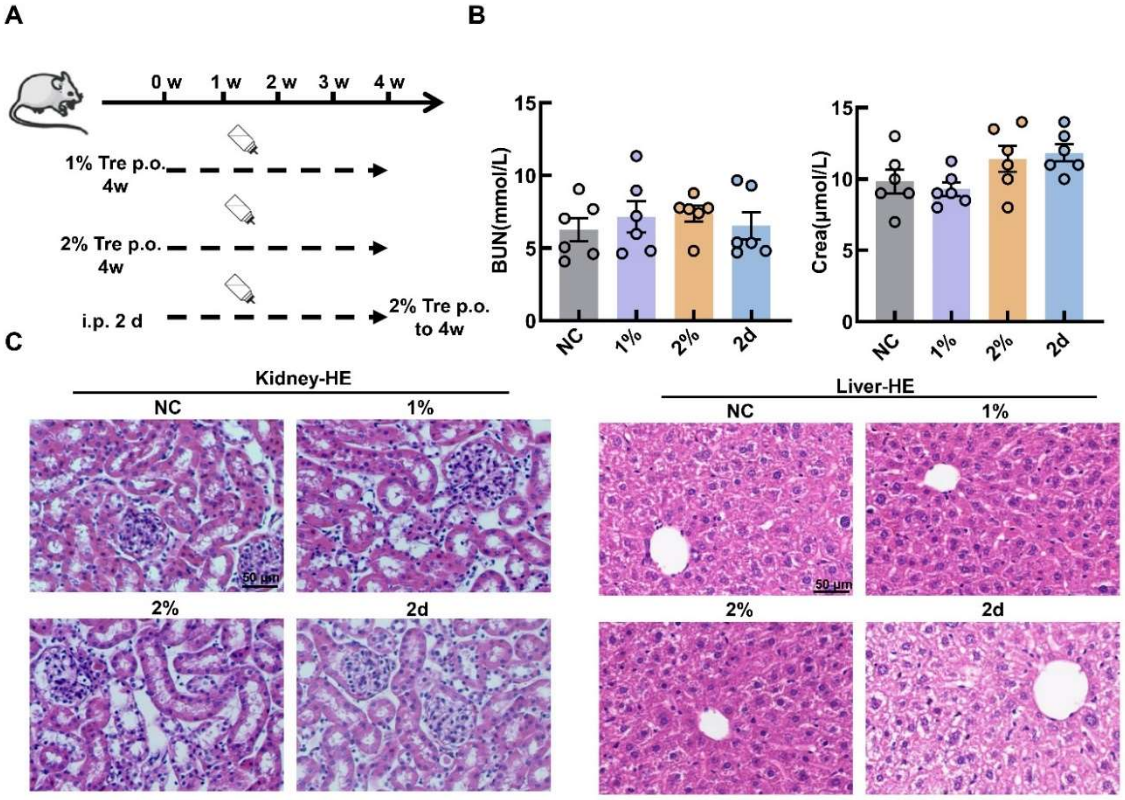

Figure S4 Trehalose has no effect on nephrotoxicity and hepatotoxicity in mice. (A) Illustration of trehalose intervention. (B) The serum levels of BUN and CREA in the mice. (C) Representative images of hematoxylin-eosin (HE) in paraffin-embedded kidney and liver sections. Scale bars, 50 μm. n = 6 mice per group were used to analyze the results. All experiments were repeated at least 3 times. (NC, normal control; 1%, mice were orally administered trehalose diluted in drinking distilled water at a final concentration of 1% w/v solution for 4 weeks; 2%, mice were orally administered trehalose diluted in drinking distilled water at a final concentration of 2% w/v solution for 4 weeks; 2d, mice were intraperitoneally injected at 1 g/kg of body weight, and 48 hours later, 2% w/v solution for up to 4 weeks).

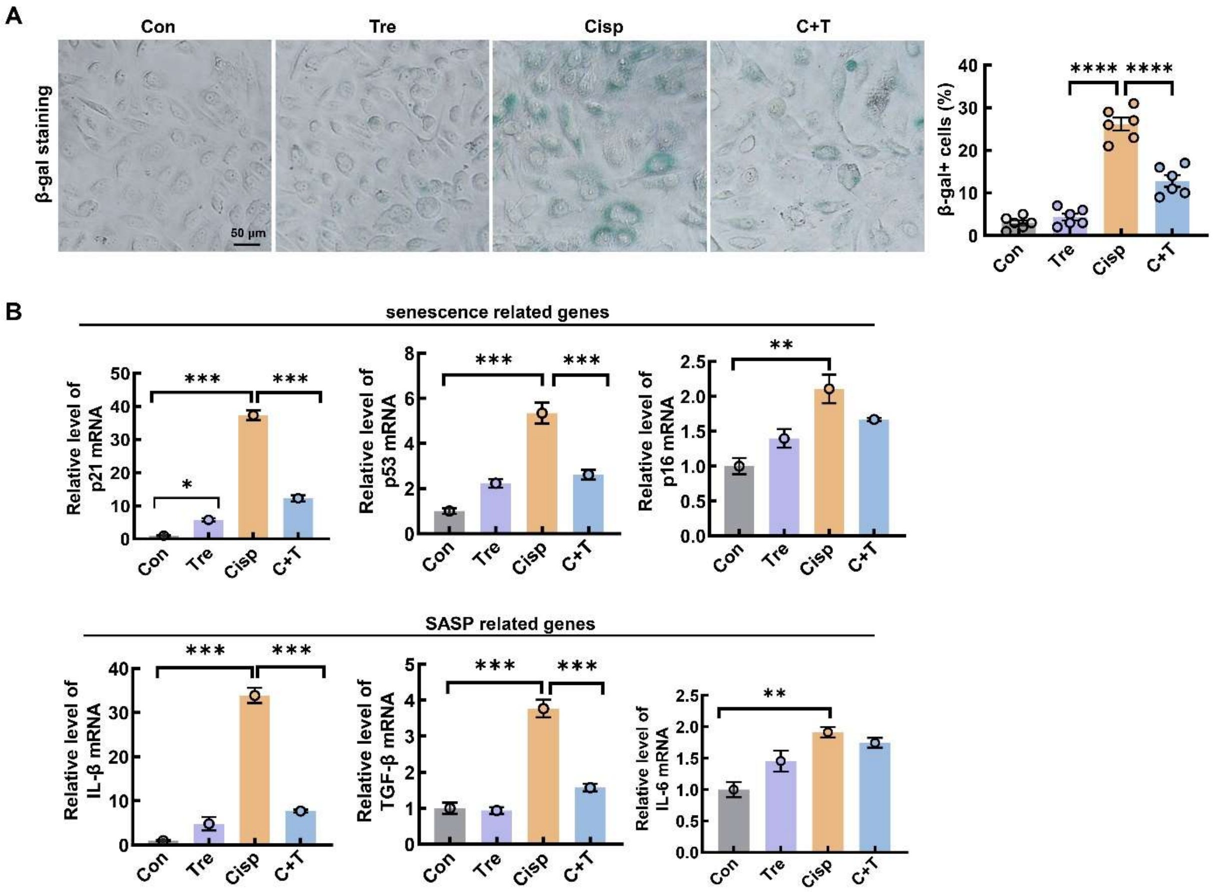

Figure S5 Trehalose ameliorates Cisplatin-induced senescence *in vitro*. (A) HK2 cells were stained for SA-β-Gal. Scale bar, 50 μm. (B) The mRNA levels of p21, p53, p16 and IL-1β, TGF-β, IL-6 in the 4 different groups. Data are shown as the means ± SD from at least three independent experiments and analyzed by one-way ANOVA with Tukey's test. *P <0.05, **P <0.01, ***P <0.001, ****P <0.0001. (Con, control; Tre, trehalose; Cisp, cisplatin; C + T, cisplatin + trehalose).

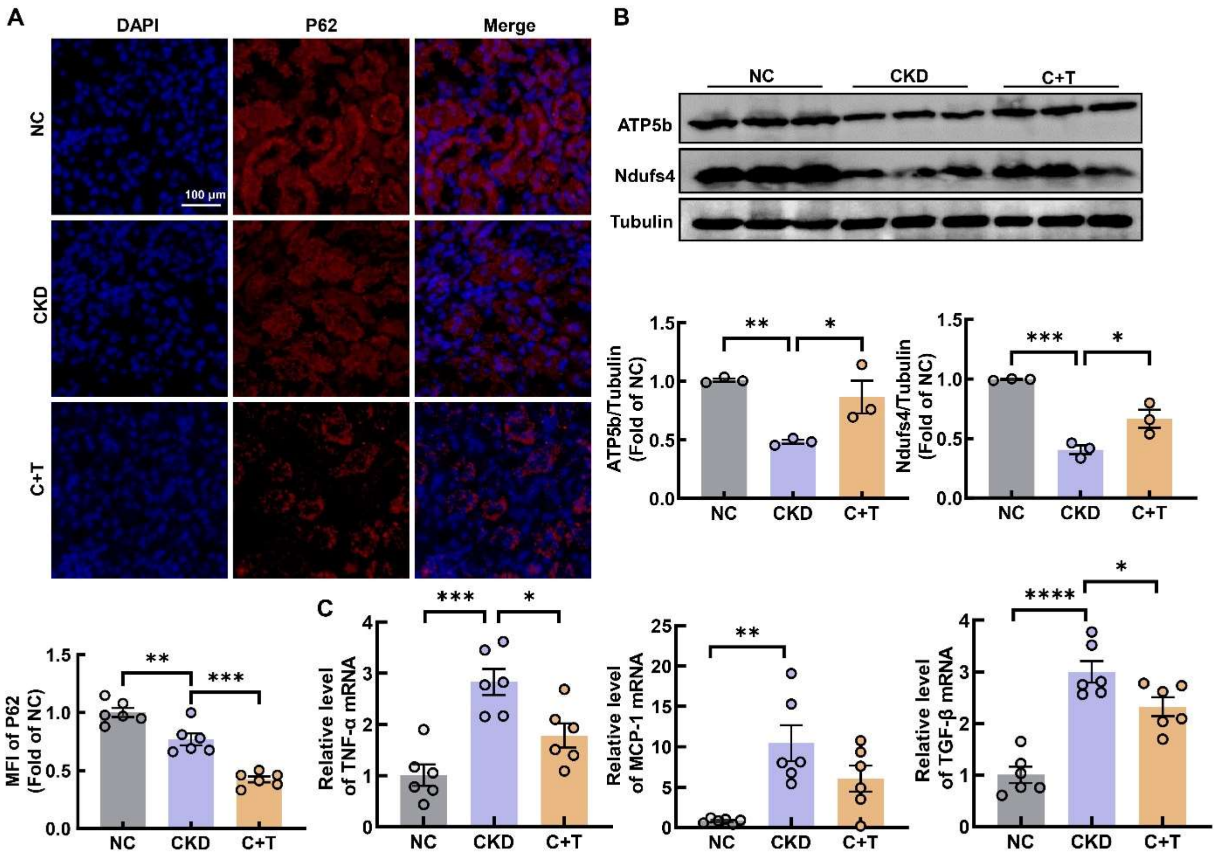

Figure S6 Post-intervention of trehalose activates autophagy and ameliorates cisplatin-induced renal injury. (A) Representative immunofluorescence images and quantification of P62 (red) in kidney sections. Scale bar, 100 μm. (B) The expression of mitochondria-related proteins (ATP5b and Ndufs4) was measured by western blotting. (C) Relative mRNA expression of SASP-associated genes, including TNF-α, MCP-1, and TGF-β in the kidney was shown. n = 6 mice per group were used to analyze the results. Data are shown as the means ± SD from three independent experiments and analyzed by one-way ANOVA with Tukey's test. *P <0.05, **P <0.01, ***P <0.001, ****P <0.0001. (NC, normal control; CKD, cisplatin-induced CKD mice; C+T, cisplatin-induced CKD mice treated with 2% w/v trehalose solution to treat CKD mice one week after the first intraperitoneal injection of cisplatin).

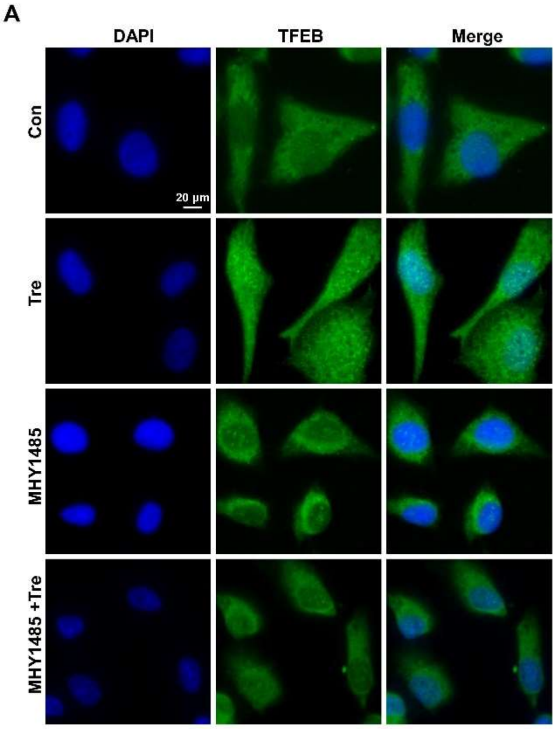

Figure S7 Trehalose accumulates in lysosomes and triggers TFEB nuclear translocation by inhibiting the mTORC1 signaling pathway. (A) Representative image of TFEB *in vitro*. HK2 cells were treated with MHY1485 (2 μM) in the presence or absence of trehalose (100 μM). Scale bar, 20 μm. (Con, control; Tre, trehalose; Cisp, cisplatin; C + T, cisplatin + trehalose; MHY1485, HK2 cells were treated with MHY1485 (2 μM) for 6 h; MHY1485 + Tre, HK2 cells were treated with MHY1485 (2 μM) for 6 h in the presence of trehalose).

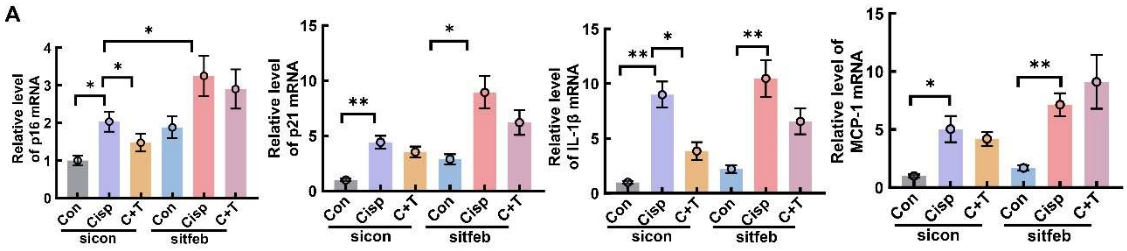

Figure S8 Silencing TFEB partially abolishes the protective effects of trehalose in cisplatin-treated HK2 cells. HK2 cells were transfected with control siRNA (sicon) or TFEB siRNA (sitfeb) for 6 h and treated with cisplatin (20 μM) in the presence or absence of trehalose (100 mM) for 72 h. **(**A) Relative mRNA expression of p16, p21, IL-1β, and MCP-1 in the kidney was shown. Data are shown as the means ± SD from three independent experiments and analyzed by one-way ANOVA with Tukey's test. *P <0.05, **P <0.01. (Con, control; Tre, trehalose; Cisp, cisplatin; C + T, cisplatin + trehalose).

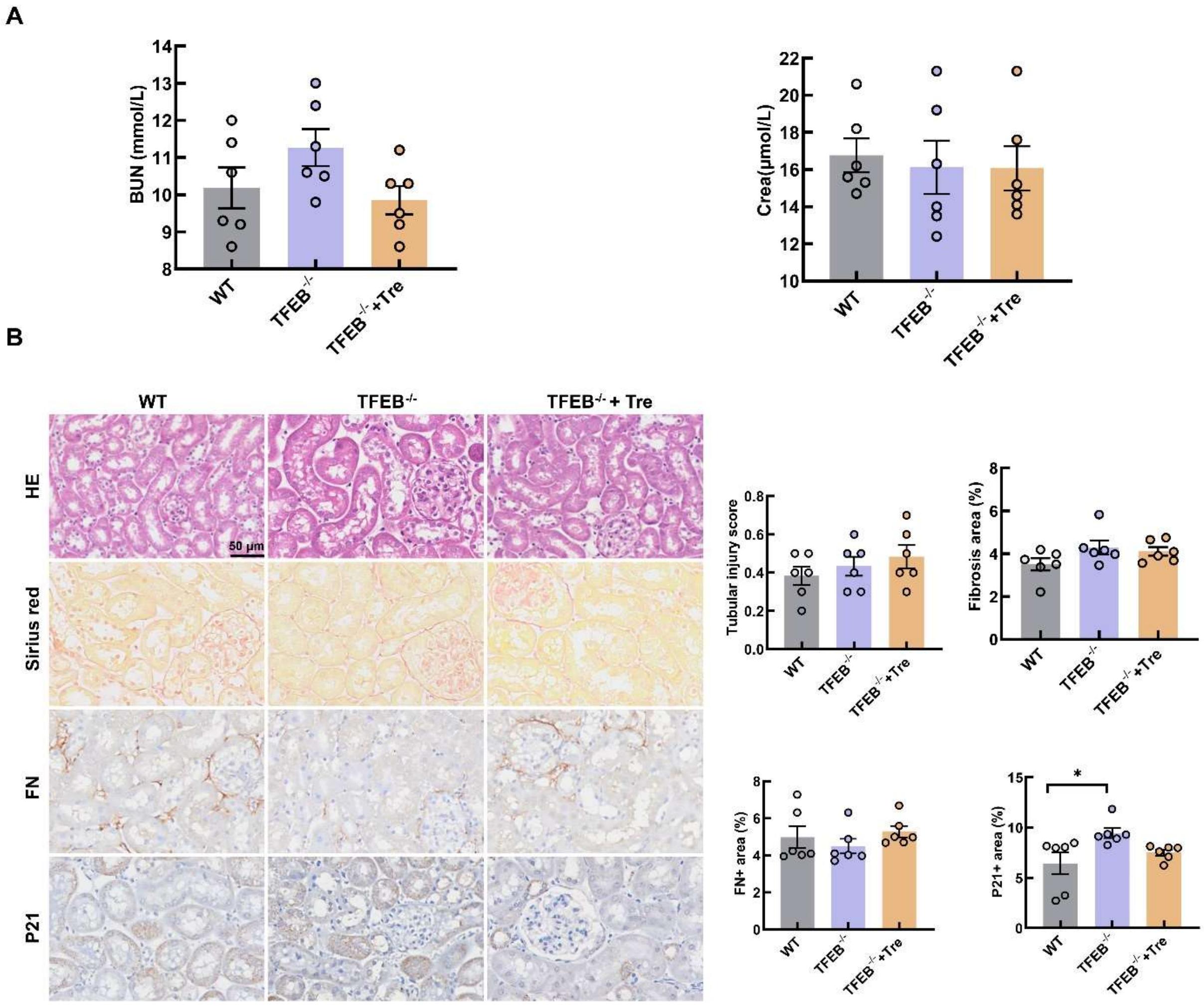

Figure S9 TECs-specific deletion of TFEB in mice has no effect on renal under physiological condition. (A) The serum levels of BUN and CREA in the mice. (B) Representative images of hematoxylin-eosin (HE) and sirius red-staining (Sirius red), immunohistochemical staining and quantification of fibronectin (FN) and P21 in paraffin-embedded kidney sections. Scale bars, 50 μm. Data are shown as the means ± SD from at least three independent experiments and analyzed by one-way ANOVA with Tukey's test. *P <0.05. (WT, wild type; TFEB$^{-/-}$, renal proximal tubule-specific TFEB mice; TFEB$^{-/-}$+Tre, renal proximal tubule-specific TFEB mice treated with trehalose).

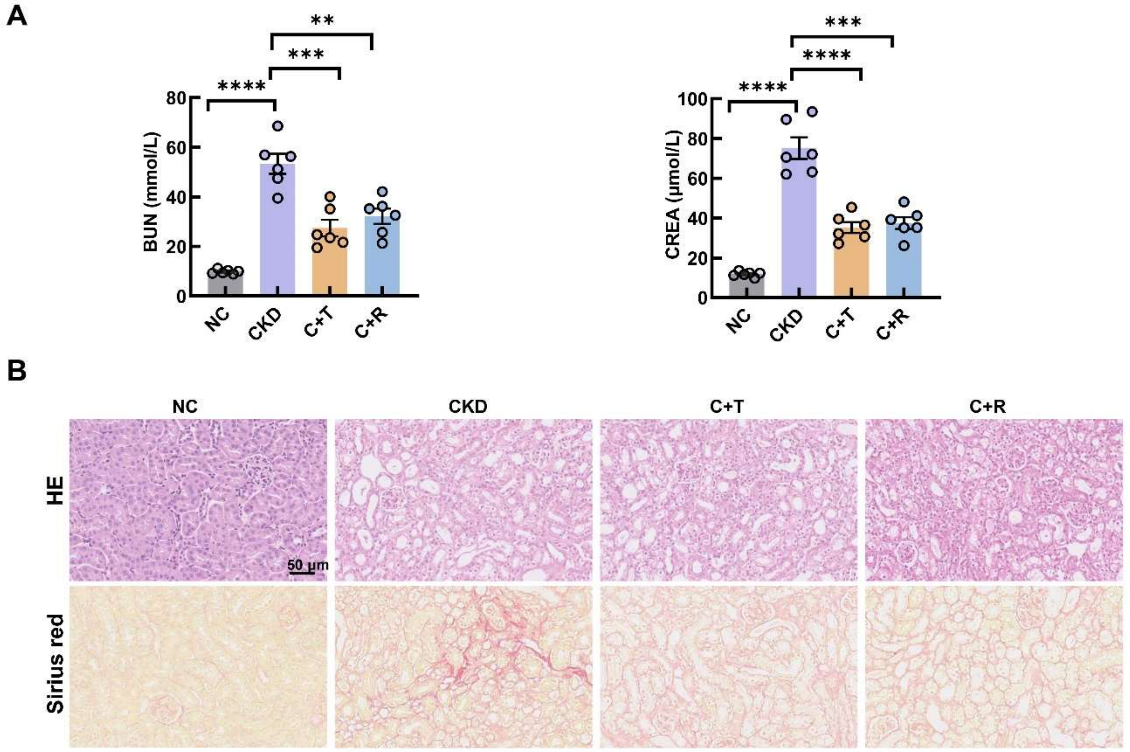

Figure S10 Trehalose and Resveratrol (RSV) relieve cisplatin-induced CKD kidney injury. (A) The serum levels of BUN and CREA in the mice. (B) Representative images of hematoxylin-eosin (HE) and sirius red-staining (Sirius red) in paraffin-embedded kidney. Scale bars, 50 μm. n = 6 mice per group were used to analyze the results. (NC, normal control; cisplatin-induced CKD mice; C+T, cisplatin-induced CKD mice treated with 2% w/v trehalose solution to treat CKD mice one week after the first intraperitoneal injection of cisplatin; C+R, cisplatin-induced CKD mice treated with resveratrol (100 mg/kg per day, i.g.)).

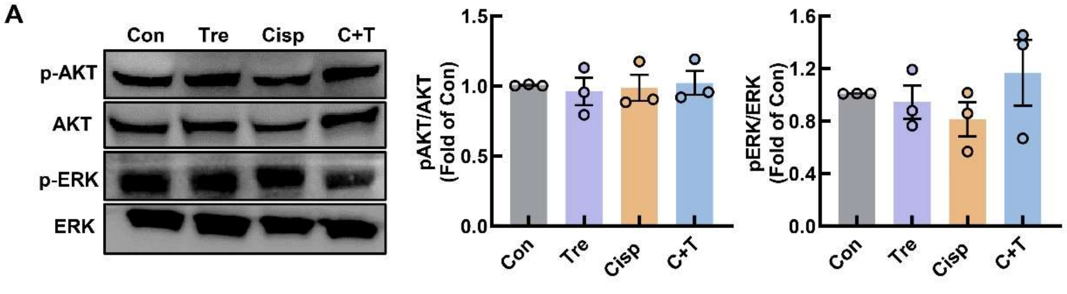

Figure S11 trehalose did not affect the expression of p-ERK and p-AKT *in vitro*. (A) The levels of p-AKT and p-ERK were measured by western blotting. Data are shown as the means ± SD from three independent experiments and analyzed by one-way ANOVA with Tukey's test. (Con, control; Tre, trehalose; Cisp, cisplatin; C + T, cisplatin + trehalose).

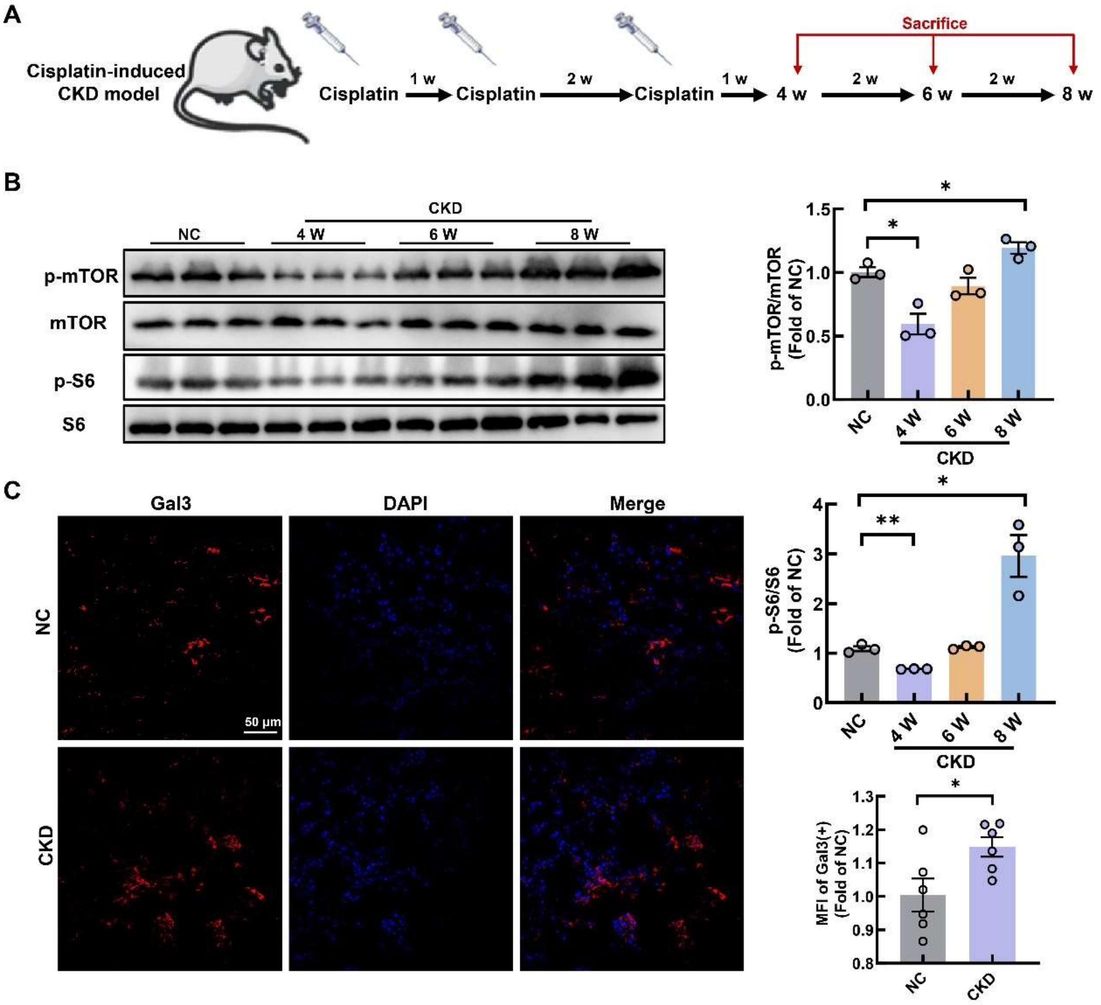

Figure S12 The tendency of mTOR is inhibited at 4 weeks and then elevated at 6-8 weeks in cisplatin-induced CKD model. (A) Illustration of cisplatin-induced CKD mice model. Mice were intraperitoneally injected with three doses of cisplatin at a concentration of 10 mg/kg at 0, 1, and 3 weeks. All the recipients were sacrificed at 4, 6 and 8 weeks after the first cisplatin injection. (B) Western blot and quantitative analyses of p-mTOR/mTOR and p-S6/S6. (C) Representative immunofluorescence images and quantification of Gal3+(red) in kidney sections. Scale bar, 50 μm. n = 6 mice per group were used to analyze the results. Data are shown as the means ± SD from at least three independent experiments and analyzed by one-way ANOVA with Tukey's test and Student's t test. *P <0.05, **P <0.01. (NC, normal control; Con, control; Tre, trehalose; Cisp, cisplatin; C+T, cisplatin + trehalose).
